# The cohesin-associated protein Wapal is required for proper Polycomb-mediated gene silencing

**DOI:** 10.1186/s13072-016-0063-7

**Published:** 2016-04-15

**Authors:** Cary Stelloh, Michael H. Reimer, Kirthi Pulakanti, Steven Blinka, Jonathan Peterson, Luca Pinello, Shuang Jia, Sergei Roumiantsev, Martin J. Hessner, Samuel Milanovich, Guo-Cheng Yuan, Sridhar Rao

**Affiliations:** Blood Research Institute, BloodCenter of Wisconsin, 8727 West Watertown Plank Road, Milwaukee, WI 53226 USA; Department of Cell Biology, Neurobiology, and Anatomy, Medical College of Wisconsin, Milwaukee, WI USA; Dana Farber Cancer Institute, Harvard School of Public Health, Boston, MA USA; Department of Pediatrics, Medical College of Wisconsin, Milwaukee, WI USA; Department of Pediatrics, Massachusetts General Hospital, Boston, MA USA; Sanford Research Center, Sanford School of Medicine, University of South Dakota, Sioux Falls, SD USA

**Keywords:** Cohesin complex, Epigenetics, Embryonic stem cells, Wapal, Polycomb complex

## Abstract

**Background:**

The cohesin complex consists of multiple core subunits that play critical roles in mitosis and transcriptional regulation. The cohesin-associated protein Wapal plays a central role in off-loading cohesin to facilitate sister chromatid separation, but its role in regulating mammalian gene expression is not understood. We used embryonic stem cells as a model, given that the well-defined transcriptional regulatory circuits were established through master transcription factors and epigenetic pathways that regulate their ability to maintain a pluripotent state.

**Results:**

RNAi-mediated depletion of Wapal causes a loss of pluripotency, phenocopying loss of core cohesin subunits. Using chromatin immunoprecipitation coupled with next-generation sequencing (ChIP-seq), we determine that Wapal occupies genomic sites distal to genes in combination with CTCF and core cohesin subunits such as Rad21. Interestingly, genomic sites occupied by Wapal appear enriched for cohesin, implying that Wapal does not off-load cohesin at regions it occupies. Wapal depletion induces derepression of Polycomb group (PcG) target genes without altering total levels of Polycomb-mediated histone modifications, implying that PcG enzymatic activity is preserved. By integrating ChIP-seq and gene expression changes data, we identify that Wapal binding is enriched at the promoters of PcG-silenced genes and is required for proper Polycomb repressive complex 2 (PRC2) recruitment. Lastly, we demonstrate that Wapal is required for the interaction of a distal *cis*-regulatory element (CRE) with the *c*-*Fos* promoter.

**Conclusions:**

Collectively, this work indicates that Wapal plays a critical role in silencing of PcG target genes through the interaction of distal CREs with promoters.

**Electronic supplementary material:**

The online version of this article (doi:10.1186/s13072-016-0063-7) contains supplementary material, which is available to authorized users.

## Background

Gene expression is regulated by the complex interplay of *cis*-acting DNA elements and *trans* acting molecules such as transcription factors (TFs). The cohesin complex plays a critical role in connecting distal *cis*-acting DNA elements to gene promoters by facilitating DNA loops [1–3]. In addition, the cohesin complex mediates sister chromatid cohesion during mitosis, ensuring proper genomic segregation [4]. In mitosis, the core cohesin subunits Smc1a, Smc3, and Rad21 are loaded onto sister chromatids by Nipbl in early G1/late telophase and off-loaded starting in prophase by Wapal [5, 6]. While the function of core cohesin subunits and accessory proteins (Nipbl, Wapal) are well established during mitosis, Wapal’s role during transcriptional regulation remains less well defined. The core cohesin subunits facilitate interactions between genes and distal *cis*-regulatory elements by “looping-out” the intervening chromatin segment [2–4, 7]. In embryonic stem cells (ESCs), the majority of cohesin-binding sites are co-occupied by CTCF, but a minority of cohesin-binding sites represent transcriptional enhancers [2, 8–10]. Depletion of cohesin core subunits by RNA interferences (RNAi) in ESCs causes differentiation secondary to either transcriptional or cell-cycle changes [2, 8, 11]. Whether Wapal participates in gene regulation through facilitating DNA loops or even binds to specific genomic regions remains unknown. However, recent studies indicate Wapal plays a role during interphase through controlling the dynamic association of cohesin with chromatin [12, 13]. However, because the precise genomic sites occupied by Wapal are unknown, it is difficult to know its precise role in regulating cohesin’s association with specific chromatin regions.

Given the critical role of cohesin in mitosis, mutations within core subunits would be expected to cause significant mitotic defect(s). A subset of patients with Cornelia de Lange Syndrome (CdLS), characterized by microcephaly, cognitive impairment, abnormal facies, and other malformations has mutations within core subunits of the cohesin complex including *Smc1a*, *Smc3*, and *Rad21* (reviewed in [14, 15]. However, these patients have sporadic heterozygous mutations, implying that complete loss of cohesin activity through null mutations is incompatible with life. Given that these mutations behave in an autosomal dominant fashion with unaffected parents, it implies that the majority of CdLS mutations occur within the parental germ cells. Surprisingly, CdLS patient samples exhibit a normal cell cycle, implying that cohesin haploinsufficiency does not cause CdLS through alterations in mitosis. Recent work has also demonstrated that heterozygous mutations in cohesin are a common (5–20 %) occurrence in patients with acute myeloid leukemia (AML) and related disorders [16, 17]. Given that AML samples rarely exhibit significant changes in chromosomal number, it again highlights that cohesin mutations likely cause disease by alterations in gene expression.

Compared to the core cohesin subunits, far less is known about the role of Wapal in transcriptional regulation. In mammals, Wapal plays a role in off-loading cohesin to prevent chromatin condensation [13], implying that Wapal likely antagonizes core cohesin subunits during transcriptional regulation. However, because the specific genomic sites occupied by Wapal are unknown, its precise role in mammalian transcriptional regulation remains unclear. In Drosophila, Wapal promotes Polycomb group silencing, although the mechanism is unclear and whether it applies to mammals is unknown [12].

How Polycomb complexes are targeted to specific genomic regions remains a critical question within epigenetics given their important role in cellular differentiation [18]. In Drosophila, Polycomb targeting is mediated by specific distal *cis*-regulatory elements (CREs) termed Polycomb response elements (PREs) [18, 19]. In contrast, mammalian PREs are rare [19], and other proposed mechanisms for PcG targeting include noncoding RNAs [20], nonspecific silencing of all transcription which must be subsequently relieved by transcriptional activators [21], interactions with other sequence-specific DNA-binding proteins such as CTCF [22, 23], or the presence of CpG islands within the promoter. Collectively, the diversity of proposed targeting mechanisms illustrates the lack of consensus within the field.

To better delineate the role of Wapal in mammalian transcriptional regulation, we have chosen murine ESCs as a model system. ESCs are unique because they possess two critical properties: *pluripotency*, or the ability to differentiate into all three primitive germ layers (mesoderm, ectoderm, and endoderm) that give rise to the embryo, and *self*-*renewal*, or the ability to propagate indefinitely in an undifferentiated state. The transcriptional and epigenetic pathways that regulate both functions have been well defined through a series of genome-wide approaches. Our study demonstrates that Wapal plays a central role in regulating transcription by assisting in Polycomb group-mediated gene silencing.

## Results

### Wapal depletion causes ESCs to differentiate

**Fig. 1:**
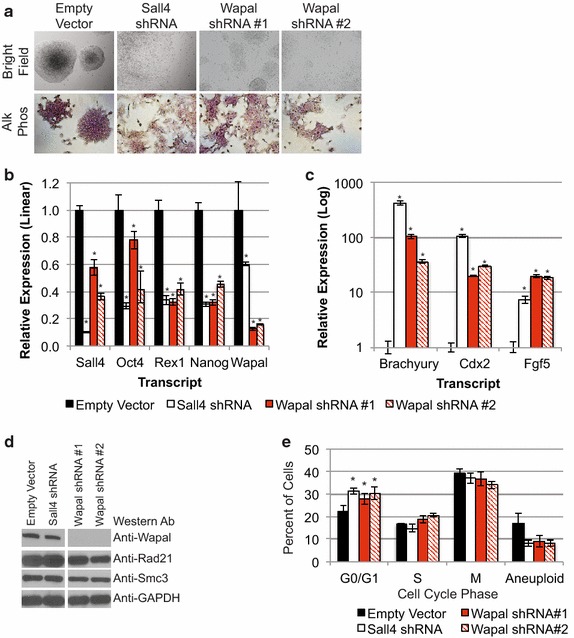
**a** ESCs were infected with lentiviruses encoding shRNAs to Sall4 and Wapal. Cells with a high multiplicity of infection (MOI) were selected by addition of puromycin for 6 days. Images were taken by bright field (BF, ×10) or after staining for the pluripotency marker alkaline phosphatase (AP, ×10). **b** Changes in pluripotency markers (Nanog, Oct4, Sall4, and Rex1) and Wapal were measured by RT-qPCR. Fold change was calculated relatively to cells infected with the empty vector and plotted linearly on the *y*-axis. *Error bars* represent SEM of at least two experiments. *Asterisk* indicates statistically significant reductions compared to empty vector (*p* value <0.05). **c** Similar to **b**, but for differentiation markers Brachyury (mesoderm), Fgf5 (ectoderm), and Cdx2 (trophectoderm). **a** Log_10_ scale for fold change is used because of strong induction of these markers. *Asterisk* indicates statistically significant increases compared to empty vector (*p* value <0.05). **d** Protein levels after depletion of Wapal or Sall4 for core cohesin subunits Rad21 and Smc3. GAPDH is shown as a loading control. Intervening, unrelated lanes were removed, but all samples were run on the same gel. **e** Propidium iodide (PI) staining was used to measure DNA levels in cells by flow cytometry. The percent of cells within each phase of the cell cycle and which exhibited abnormal DNA content (<2N or >4N) is shown. The only statistically significant difference was in the fraction within G0/G1 after depletion of Sall4 or Wapal as compared to empty vector (*asterisk* indicates *p* value <0.05)

Depletion of cohesin complex core subunits (Smc3, Smc1a, Scc1/Rad21) causes a loss of pluripotency when depleted in ESCs [2, 8, 11]. To examine whether loss of Wapal had a similar effect, we depleted Wapal in ESCs using RNAi. We infected ESCs with puromycin-resistance-encoding lentiviruses containing short-hairpin RNAs (shRNAs) to deplete Wapal. As a positive control, we depleted the pluripotency TF Sall4 with a single shRNA; a second shRNA to Sall4 gives similar results [24, 25]. Depletion of both Wapal and Sall4 induced a loss of compact, spherical colonies indicative of differentiation 6 days after infection (Fig. 1a, top panels). Surface expression of the pluripotency marker alkaline phosphatase was partially reduced and highlights the significant morphological changes that occurred following Wapal depletion (Fig. 1a, bottom panels). To quantitate the changes in pluripotency, we used Cas9-mediated genomic editing to create an ESC line with an internal ribosomal entry site:enhanced green fluorescent protein reporter (*IRES:EGFP)* cassette inserted into the *Oct4* locus, allowing EGFP expression to be a surrogate marker for pluripotency. The same approach has been used to generate a flow cytometry-based assay to quantitate changes in pluripotency [26, 27]. After 6 days of puromycin selection, we observed a statistically significant reduction in the mean fluorescent intensity (MFI) of the GFP peak after depleting with shRNAs to Sall4 or Wapal (shRNA #2, Additional file 1: Figure S1a). Wapal shRNA #1 caused a reduction in the MFI but did not reach statistical significance (*p* value <0.08). Taken together, the changes in cell morphology and alkaline phosphatase activity and decrease in *Oct4:IRES:EGFP* expression indicate that Wapal depletion induces a loss of pluripotency and subsequent differentiation of ESCs. Similar results have been observed by other groups when depleting Smc3, Smc1a [2], or Rad21 [8], indicating that depletion of Wapal phenocopies the loss of core cohesin subunits on ESC pluripotency.

We further examined the effects of each shRNA on known pluripotency markers (Fig. 1b). Both shRNAs induced a strong knockdown of Wapal by greater than 80 %. Wapal knockdown was associated with a significant reduction in the expression of pluripotency markers such as Oct4, Rex1, and Nanog. To examine whether differentiation markers are increased in expression after Wapal depletion, we screened a panel of lineage-specific markers including Cdx2 (trophectoderm), Brachyury (mesoderm), and Fgf5 (ectoderm). As shown in Fig. 1c, depletion of Wapal is associated with a dramatic increase in each marker, indicating differentiation into these lineages. Collectively, these data indicate that Wapal is required for ESC pluripotency. To determine whether Wapal depletion induces differentiation through indirect effects on other members of the cohesin completion, we performed Western blots to assess the levels of two core subunits of the cohesin complex, Rad21 and Smc3 (Fig. 1d). Wapal depletion had minimal effect on Rad21 or Smc3 protein levels, indicating that the loss of pluripotency is unlikely to be secondary to altered expression of other core cohesin subunits.

Given the role of Wapal in off-loading cohesin from sister chromatids at the end of mitosis, we assessed the cell cycle to determine whether Wapal depletion induced aneuploidy, which could cause a loss of pluripotency. We measured DNA content by propidium iodide (PI) staining in ESCs 48 h after Wapal depletion and assessed the percent of cells in different stages of the cell cycle or that exhibited aneuploidy (Fig. 1e). We chose an earlier time point than our differentiation experiments because subtle changes in the cell cycle early in the process could be responsible for differentiation. Depletion of neither the pluripotency TF Sall4 nor Wapal increased the number of aneuploid cells. We saw a slight increase in the number of cells in G0/G1 when we depleted either Sall4 or Wapal, but little change in the percentage of cells in M or S phase. Collectively, these data indicate that Wapal depletion does not dramatically alter the cell cycle or promote aneuploidy in ESCs. Thus, the loss of pluripotency is unlikely to be caused by a mitotic defect or aneuploidy.

To ensure that the pluripotency changes were not secondary to off-target effects of our shRNAs, we generated an ESC line expressing an epitope (v5)-tagged version of full-length Wapal immune to shRNA #2 by mutating every third base in each codon within the shRNA recognition site. This strategy prevents recognition by the shRNA without altering the linear amino acid sequence. Infection with shRNA #2 on wild-type cells caused a loss of pluripotency as assessed by cell morphology that was completely rescued in cells expressing the full-length Wapal immune to the shRNA (FL Wapal Imm, Additional file 1: Figure S1b). In addition to rescuing the morphology, expression of FL Wapal Imm rescued the changes in pluripotency markers such as Nanog and Oct4 (Additional file 1: Figure S1c) and suppressed the induction of differentiation markers such as Brachyury, Bmp2, Cdx2, and Fgf5 (Additional file 1: Figure S1d). We assessed protein levels of a subset of markers (Additional file 1: Figure S1e), which were decreased (Nanog) or increased (Brachyury) after Wapal depletion. Expression of FL Wapal Imm blocked the protein changes in Nanog and Brachyury observed after Wapal depletion. Collectively, the phenotypic rescue by FL Wapal Imm indicated that the loss of pluripotency we observed is secondary to Wapal depletion and not an off-target effect of RNAi.

### Wapal preferentially occupies genomic regions with other cohesin proteins

To identify genomic regions occupied by Wapal, we screened for antibodies which met ENCODE criteria [28] for chromatin immunoprecipitation coupled with next-generation sequencing (ChIP-seq). All antibodies tested were unsuitable for ChIP-seq; therefore, we turned to a metabolic labeling approach to tag Wapal with biotin [24, 29]. As a first step, we determined the level of expression of the tagged Wapal constructs stably expressed in ESCs. By Western blot (Additional file 2: Figure S2a), the total levels of Wapal protein were unchanged between two clones expressing tagged Wapal (FB Wapal) versus a control cell line expressing the biotin ligase BirA in the absence of tagged Wapal. As a positive control, we included a previously published cell line expressing a tagged Nanog (FB Nanog, [29]). Thus, FB Wapal is expressed at subendogenous levels, consistent with our previous published data, and meets ENCODE criteria for ChIP-seq [24, 28, 29]. We performed ChIP-seq on two independent FB Wapal clones and subtracted genomic regions also present in our BirA-only control for downstream analyses (Additional file 2: Figure S2b, see “Methods” section for details). Five Wapal-binding sites were verified by ChIP-qPCR at genomic sites co-occupied by Wapal and other subunits of the cohesin complex (Additional file 2: Figure S2c and below).

**Fig. 2:**
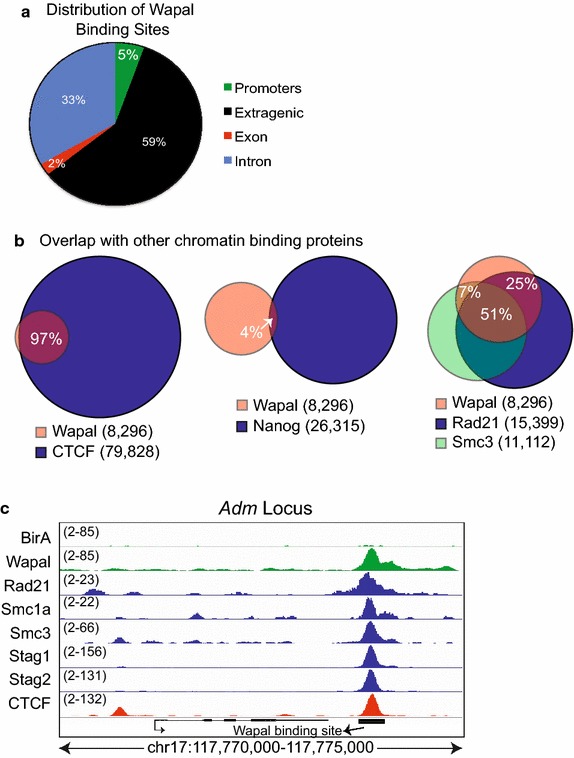
**a** The genomic distribution of Wapal-binding sites. **b** Overlap of Wapal-binding sites with CTCF, Nanog, or core subunits of the cohesin complex Smc3 and Rad21. Percentages listed indicate the area(s) of overlap of Wapal-binding sites with a specific factor. **c** ChIP-seq tracks for *Adm* to demonstrate the binding of Wapal, CTCF, and cohesin components. The *y*-*axis* represents the # of ChIP-seq tags recovered for a given genomic bin. The range for each track is displayed to the right of track name. A lower threshold of 2 was set for all tracks to minimize background. In all cases, the *y*-*axis* for BirA and Wapal are the same to demonstrate binding specificity

As a first step, we analyzed all Wapal-binding sites to determine their distribution within the genome. The majority (≈60 %) were in extragenic regions, and only a small percentage (5 %) were within gene promoters (Fig. 2a). Wapal is known to directly interact with the core cohesin subunit Rad21 during mitosis to facilitate off-loading cohesin [5, 30]. We have also demonstrated that Wapal interacts with the pluripotency-associated transcription factor Nanog [29]. CTCF, which has pleiotropic functions in regulating transcription, directly interacts with core cohesin subunits (Smc3, Rad21, and Stag1/2) to stabilize DNA loops required for long-range chromatin interactions, although a direct CTCF–Wapal interaction has not been reported [3, 31, 32]. We overlapped our Wapal-binding sites with those of Rad21, Nanog, and CTCF to determine whether they co-occupy the same genomic regions (Fig. 2b). We were surprised that Wapal and Nanog showed minimal overlap (4 %) with each other, implying that they bind to distinct genomic regions. In contrast, Wapal showed a high degree of overlap with CTCF (97 %). Genomic sites occupied by Wapal showed a strong overlap with core cohesin subunits (75 % overlap between Wapal and Rad21, Fig. 2b, c). Collectively, this indicates that Wapal co-occupies DNA elements with core cohesin subunits and CTCF. Importantly, given that CTCF recruits core cohesin subunits to DNA elements [3, 33], it is likely that Wapal is recruited to these sites in conjunction with other cohesin subunits rather than through a direct Wapal–CTCF interaction (Additional file 3: Figure S3).

### Wapal does not affect cohesin binding at co-occupied genomic sites

**Fig. 3:**
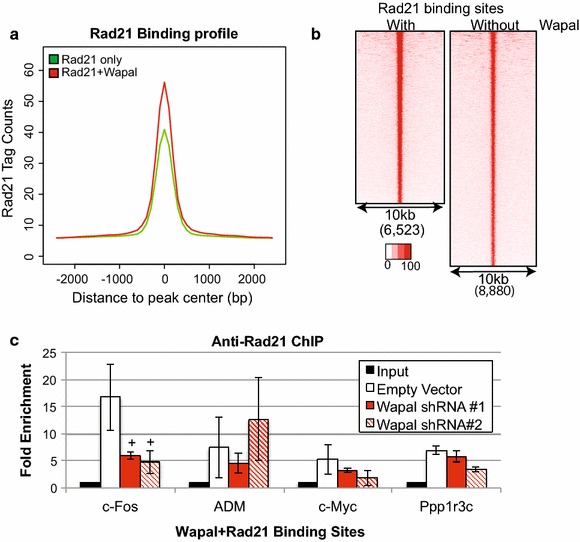
**a** The ChIP-seq tag densities of Rad21 were compared at genomic sites occupied with (*red*) or without (*green*) Wapal in a 2.5 kb window around peak center. **b** ChIP-seq tag densities for each Rad21 binding site within the genome is visualized as individual rows, with the *color scale* indicating a relative linear gradient from 0 to maximal (100 %) tag values. Two different plots were generated based upon the binding with (*left*) or without (*right*) Wapal. Row order is from lowest binding to highest binding. **c** Wapal was depleted with two, independent shRNAs for 48 h and genomic occupancy of Rad21 measured by ChIP-qPCR. The genomic sites (*x*-*axis*) are all co-occupied by both Wapal and Rad21. All sites demonstrated statistically significant enrichment in the empty vector sample compared with input (*p* value <0.05). *Plus symbol* indicates a statistically significant reduction from empty vector (*p* value <0.05)

Recently, it has been demonstrated that Wapal antagonizes cohesin binding to chromatin during interphase, similar to its role during mitosis [5, 12, 13]. However, because Wapal’s genomic occupancy was unknown, previous studies were performed with bulk analysis of cohesin binding to chromatin and not at specific genomic sites [12, 13]. We examined all loci occupied by Wapal in combination with core cohesin subunits (Rad21, Smc1a, and Smc3) to determine whether Wapal-occupied genomic sites display lower levels of cohesin binding. Genomic sites occupied by Wapal consistently displayed higher ChIP-tag densities for Rad21, Smc3, or Smc1a (Fig. 3a, b; Additional file 4: Figure S4) as compared to sites where these factors bound without Wapal. To confirm this result directly, we measured the chromatin occupancy of Rad21 after Wapal depletion by ChIP-qPCR at a handful of loci co-occupied by both Wapal and Rad21 (Fig. 3c). If Wapal promotes cohesin of off-loading, Wapal depletion should increase Rad21 occupancy as measured by ChIP-qPCR. We did not observe a statistically significant increase in Rad21 binding at four loci as compared to the empty vector. Collectively, these data would indicate that Wapal depletion did not promote off-loading cohesin at genomic they co-occupy.

### Wapal depletion is associated with the derepression of Polycomb targets

To assess transcriptome changes after Wapal depletion, we hybridized cDNA generated from ESCs 48 h post-lentiviral infection to microarrays. We chose a short time frame to minimize differentiation effects and enrich for target genes. Wapal-depleted cells showed no changes in morphology as compared to empty vector at this early time point (Additional file 5: Figure S5a). To assess whether pluripotency and differentiation markers are altered at this early time point, we performed reverse transcription coupled with quantitative PCR (RT-qPCR) for various markers that exhibited changes after longer periods (6 days) of Wapal depletion. We observed minimal changes in Nanog, Rad21, or Smc3 (Additional file 5: Figure S5b). Differentiation markers Brachyury, Bmp2, and Cdx2 did not increase. Thus, pluripotency is not compromised, and the cells do not differentiate significantly 48 h after Wapal depletion.

**Fig. 4:**
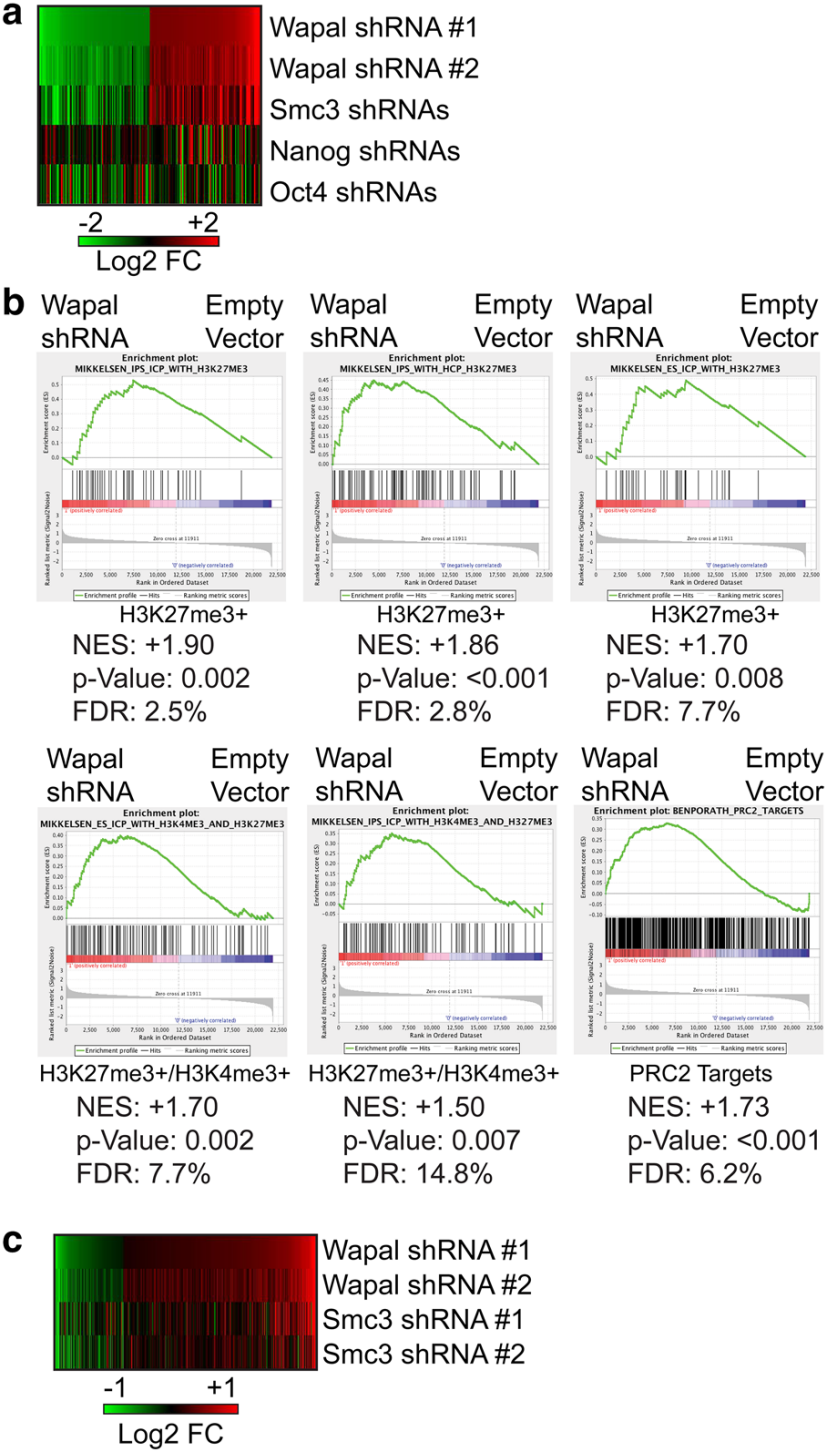
**a** The 100 probes most upregulated (*red*) or downregulated (*green*) with shRNA #1 to Wapal were identified and the Log2 fold change (Log2 FC) calculated for Wapal shRNA #2, Smc3 shRNAs, and from published studies in which the pluripotency-associated TFs Nanog or Oct4 were depleted by siRNAs. Log2 FC is displayed, with the color scheme indicated. **b** GSEA was used (see “Methods” section for details) to identify pathways globally dysregulated by Wapal depletion. Six gene sets from pluripotent cells which all exhibited substantial enrichment in Wapal-depleted samples are shown. Positive normalized enrichment score (NES) indicates that the gene set is enriched in Wapal-depleted samples as compared to samples infected with the empty vector. False discovery rate (FDR < 25 %) and *p* value <0.01 were used to determine statistically significant changes in gene set expression. **c** ChIP-seq datasets were used to generate a list of high-confidence targets of Polycomb-mediated gene silencing by the presence of both H3K27me3 and H2Aub1 in their promoters (1455 genes). For each gene, the expression changes after Wapal or Smc3 depletion were measured by microarray. For genes with multiple probes, maximal probe deviation was used. Genes that showed minimal change in their expression after Wapal depletion or the two shRNAs did not demonstrate similar changes were eliminated because of possible off-target effects. Log_2_ fold change is displayed, with a *color scheme* shown below

As further proof that the gene expression changes we observed were unique to Wapal depletion and not secondary to differentiation effects, we downloaded and reanalyzed microarray data from ESCs induced to differentiate by depleting two pluripotency-associated transcription factors, Nanog or Oct4 [34]. We compared the 100 genes either upregulated or downregulated by Wapal depletion and mapped changes at the same genes after Nanog or Oct4 depletion, which are known to induce ESCs to differentiate. We observed very little concordance between the gene expression changes seen with Wapal and Nanog/Oct4 depletion, implying that at least initially the gene expression changes are distinct, even though the end result (ESC differentiation) is the same (Fig. 4a). We also wondered whether Wapal depletion would affect the transcriptome differently than loss of a core subunit of the cohesin complex. We depleted Smc3 using two independent shRNAs and compared the gene expression changes with those after Wapal depletion. There was a striking similarity in the gene expression patterns when comparing Wapal- and Smc3-depleted cells (Fig. 4a), implying that the transcriptome changes were very similar. Collectively, our data suggest that Wapal depletion induces transcriptome changes similar to Smc3 loss, but distinct from depletion of Nanog or Oct4. Thus, although depletion of Wapal/Smc3 and Nanog/Oct4 all causes ESCs to differentiate, they are likely through distinct mechanisms.

To identify pathways or groups of genes dysregulated by Wapal depletion, we utilized gene set enrichment analysis (GSEA) that measures whether a defined set of genes shows a statistically significant, concordant difference between two biological conditions [35]. We screened 3395 different curated gene sets generated by perturbing mammalian cell systems by chemical, RNAi, or genetic means to identify Wapal-induced gene expression changes. We were not surprised that a large number of gene sets (Additional file 6: Table S5) were either upregulated (181) or downregulated (218) after Wapal depletion in a statistically significant manner (*p* value <0.01 and false discovery rate-FDR <25 %). As a first step, we focused on gene sets derived from pluripotent cells as being more pertinent to our analyses. In particular, we noted that Polycomb-marked genes were derepressed after Wapal depletion in pluripotent cells [36–38]. We observed a total of six different Polycomb target gene sets derepressed following Wapal depletion (Fig. 4b) with a high degree of statistical significance. These data are consistent with existing literature in Drosophila that Wapal promotes Polycomb-mediated gene silencing [12]. Two gene sets significantly downregulated after Wapal depletion is described as “core” modules of the pluripotent state, indicating that genes required to maintain ESCs in an undifferentiated state exhibit decreased expression (Additional file 5: Figure S5c). Thus, Wapal may play a role in silencing of Polycomb target genes in mammalian ESCs.

To validate whether Wapal depletion causes a derepression of PcG-silenced genes, we downloaded two different, independent gene sets where PcG target genes were identified [39, 40]. Both of these gene sets demonstrated a statistically significant upregulation after Wapal depletion (Additional file 5: Figure S5d), indicating that the gene sets demonstrated increased expression after Wapal depletion. As a second, independent confirmation, we downloaded and reanalyzed ChIP-seq data for the PRC1 mark of ubiquitination of histone 2A (H2AUb1) [39] and the PRC2 mark trimethylation of histone 3 on lysine 27 (H3K27me3) [41]. Genes marked by either PRC1 (H2Aub1) or PRC2 (H3K27me3) in their promoter showed a global increase after Wapal depletion, again consistent with a role for Wapal in the silencing of PcG genes (Additional file 5: Figure S5e). Given that GSEA analyzes all genes as a group, we performed a differential expression analysis to address whether PcG-silenced genes are globally derepressed or a subset of genes was skewing our analysis. To accomplish this, we generated a high-confidence list of PcG-marked genes by the presence of both H3K27me3 and H2Aub1 in their promoters. Next, we assessed transcriptional changes after Wapal depletion for each gene (Fig. 4c) and observed a 3:1 ratio of genes upregulated versus downregulated after Wapal depletion. A similar pattern was seen after Smc3 depletion (Fig. 4c). Collectively, our data indicate that Wapal depletion is associated with derepression of PcG-silenced genes, similar to the gene expression phenotype observed in Drosophila [12].

### Wapal depletion selectively reduces Ring1b occupancy at PcG-marked genes

Recent studies in Drosophila [42] indicate that the cohesin complex plays a critical role in mediating gene expression by sequestering the PRC1 complex at active genes, thereby preventing PRC1 from inducing gene silencing. To address whether this is also occurring in mammalian cells, we performed ChIP-seq with an antibody to Ring1b, a core subunit of the PRC1 complex(es), which mediates histone ubiquitination, after Wapal depletion. We saw robust binding of Ring1b at PcG-silenced genes (1455 total), which decreased after Wapal depletion (Additional file 7: Figure S6a), consistent with Wapal playing a role in PcG targeting. To examine whether Wapal depletion induced a broad redistribution of Ring1b genomic occupancy, we examined a similar number (1455) of high- and low-expression genes from our microarray data. We saw virtually no binding of Ring1b to high-expression genes, which was essentially unchanged after Wapal depletion (Additional file 7: Figure S6b). We saw low-level binding of Ring1b to low-expression genes, which again remained unchanged after Wapal depletion. Collectively, our data imply that loss of Wapal induced the changes in Ring1b occupancy at PcG-marked genes alone and does not induce redistribution across high- and low-expressed genes.

We also hypothesized that if Ring1b genomic changes were secondary to differentiation of ESCs, rather than genomic occupancy induced by Wapal, we should see altered Ring1b occupancy at genes either upregulated or downregulated during differentiation. To address this, we identified the top 1000 genes upregulated or downregulated by depletion of Nanog or Oct4 and assessed changes in Ring1b occupancy (Additional file 7: Figure S6c). We found that both sets of genes showed minimal enrichment for Ring1b and minimal change in Ring1b occupancy after Wapal depletion. Given this finding, it is highly unlikely that the transcriptome and epigenomic changes we observe after Wapal depletion are secondary to ESC differentiation.

### Wapal is required for maintenance of the PRC2 mark H3K27me3 at PcG-silenced genes

**Fig. 5:**
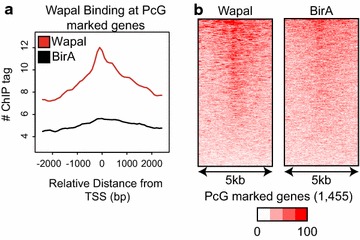
**a** A total of 1455 Polycomb-marked (H3K27me3^+^/H2Aub1^+^) genes were identified and the ChIP-seq tag densities measured for Wapal and the negative control cell line expressing BirA alone. A 5 kb window around the TSS of each gene is shown along the *x-axis*. **b** Similar to 3B, *each row* represents an individual Polycomb-marked gene, and the relative binding of Wapal and BirA is shown for each. Row order is determined by binding intensity for Wapal. *Color gradient* is a relative linear gradient from no binding (0 %) to maximal binding (100 %)

Given that PcG-mediated gene silencing occurs predominantly through histone modifications deposited within promoters, we were surprised that Wapal depletion caused PcG-marked gene derepression, given that only a small fraction (5 %) of Wapal-binding sites are within promoters. In fact, only 3 % of the high-confidence PcG-marked genes (1455) we identified had a Wapal-binding site within 2 kb of the transcriptional start site (TSS, Additional file 8: Table S1). In Drosophila, PcG genomic targeting is mediated by distal CREs termed Polycomb response elements (PREs), but in mammals PREs are rare [18, 19, 43]. Additionally in Drosophila, cohesin depletion decreases long-range interactions between a PRE and target gene(s) [42]. Given this, we hypothesized that Wapal may bind at distal CREs but through higher-ordered chromatin structures be brought into close physical proximity of gene promoters. In this case, ChIP-seq signals can often be detected at a gene’s promoter, although the binding is not substantial enough to give a positive enrichment through peak-calling algorithms. This type of binding, referred to as indirect binding, has been extensively studied by one group [44] and identifies long-range chromatin interactions critical for proper gene expression. As a first test, we examined the binding of Wapal to PcG-marked genes (Fig. 5) and observed that Wapal exhibited elevated binding at these gene promoters as compared with the BirA control. We also analyzed the subset of PcG-marked genes derepressed after Wapal depletion (Additional file 9: Figure S7a, b) and found a similar result, indicating that Wapal binding is enriched at this subset of promoters as well. A very similar pattern of binding was seen for Smc3 at PcG-marked genes (Additional file 9: Figure S7c, d), indicating that Wapal and core cohesin components behave similarly in terms of binding to PcG-marked genes in ESCs.

To perform a candidate gene analysis, we identified six PcG-marked genes derepressed after Wapal depletion for further analysis (Additional file 10: Figure S8a). Of these six genes, two (*Snai2* and *Ppp1r3c*) had combined Wapal-/CTCF-binding sites outside the promoter but within 10 kb of the TSS. In both cases (Additional file 10: Figure S8b), a clear peak of Wapal binding near the TSS could be seen, but did not reach the threshold to be identified as a binding site during peak calling. Among the four other genes (*HoxD1*, *Sox11*, *Twist1*, and *Lefty1*), we were unable to identify a Wapal-binding site within 10 kb of the gene, but nonetheless noted evidence of Wapal enrichment around the TSS (Additional file 11: Figure S9). Collectively, this indicates that Wapal binds predominantly at distal CREs, but nonetheless displays binding at PcG-silenced genes.

**Fig. 6:**
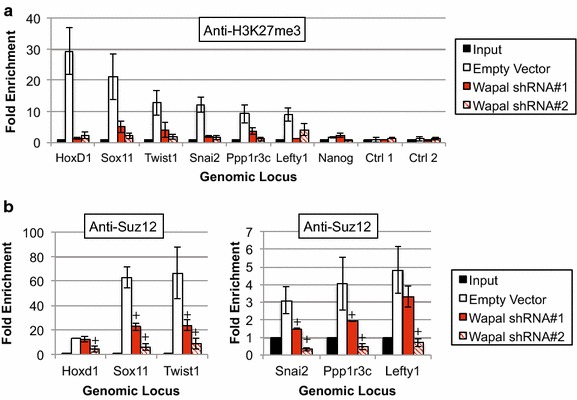
**a** ChIP-qPCR was performed at six genomic loci (*HoxD1*, *Sox11*, *Twist1*, *Snai2*, *Ppp1r3c*, and *Lefty1*) all derepressed after Wapal depletion with an antibody specific to H3K27me3. *Nanog*, which is well expressed in ESCs, is used as a negative control. Two additional negative controls (Ctrl 1 and Ctrl 2) are included—genomic regions within gene deserts. Genomic coordinates amplified by each primer set are given in Additional file 15: Table S4. *Asterisk* indicates a statistically significant difference from input (*p* value <0.05). **b** to **a**, but with an antibody to a core subunit of the PRC2 complex, Suz12. *Asterisk* indicates a statistically significant difference from input. *Plus symbol* indicates a statistically significant reduction in Suz12 enrichment from the empty vector control

To determine whether Wapal depletion caused global alterations in the activity of either PRC1 or PRC2 complexes, we measured protein levels by Western blotting with antibodies specific for PRC marks (H2Aub1 and H3K27me3) along with core subunits Ring1B (PRC1), Ezh2 (PRC2), and Suz12 (PRC2). In each case, we saw trivial changes in the protein expression of the histone marks and core PRC subunits (Additional file 12: Figure S10). This was surprising given our derepression data and implies that global PcG activity is unaffected by loss of Wapal. To further study this issue, we performed ChIP-qPCR at six derepressed genes (*HoxD1*, *Sox11*, *Twist1*, *Snai2*, *Ppp1r3c*, and *Lefty1*) 48 h after depleting Wapal with two shRNAs (Fig. 6). Primers to the *Nanog* promoter or two intergenic regions (Ctrl1, 2) were included as a negative control; we observed no enrichment at these loci using a nonspecific IgG (data not shown). Given the results of our GSEA, we focused on the PRC2 mark H3K27me3. In addition, per the classical models of PcG action in mammals PRC1 activity is preceded by PRC2-mediated deposition of H3K27me3 [18]. Strikingly, we found a substantial loss in the PRC2 mark H3K27me3 (Fig. 6a). Given these findings, we examined the genomic occupancy of Suz12, a core subunit of the PRC2, at these same genomic elements (Fig. 6b). We saw a statistically significant decrease in Suz12 occupancy at four loci with both shRNAs, indicating that the loss of H3K27me3 is associated with reduced occupancy by PRC2. The remaining two loci (*HoxD1* and *Lefty1)* showed a statistically significant reduction in Suz12 occupancy with only one shRNA, but the trend was consistent with the second shRNA. Collectively, these data demonstrate that Wapal depletion is associated with decreased PRC2 occupancy and activity at derepressed genes.

### Wapal is required for proper DNA looping of a distal CRE in the *c*-*Fos* locus

Given our findings of derepression of genes marked by PRC2 after Wapal depletion with binding of Wapal to their promoters, we hypothesized that Wapal may assist with recruiting distal CREs into gene promoters to mediate PRC2 recruitment. There is strong evidence that CTCF, which overlaps with 97 % of Wapal-binding sites (Fig. 2b), plays a role in Polycomb targeting. First, CTCF is required for Polycomb-mediated imprinting of the *Igf2/H19* locus in murine cells and directly interacts with Suz12 [22, 45]. Second, CTCF plays a central role in mediating developmental regulation of the *Hox* clusters, which are classically silenced in non-expressed cell types through a PcG-dependent mechanism [46–49]. Third, our prior work indicates that CTCF-binding sites can serve as predictors of Polycomb-mediated gene silencing in ESCs [40]. Thus, there is strong evidence in the literature that CTCF either directly recruits the PRC2 complex to silence gene expression or plays an indirect role by mediating higher-order chromatin structures.

To address whether Wapal plays a role in facilitating chromatin interactions, we focused on the *c*-*Fos* locus for several reasons. First, it is silenced in pluripotent cells and derepressed after Wapal depletion (Additional file 13: Figure S11a and [50]). Second, approximately 14 kb downstream of the gene is a combined Wapal-/CTCF-binding site, making it the closest genomic site capable of regulating c-*Fos* expression in-*cis.* Third is that the *c*-*Fos* locus is relatively isolated, with no other protein-coding loci within 100 kb on either side, making it less likely this Wapal-/CTCF-binding site is acting on other nearby genes.

As a first step, we verified that CTCF mRNA levels were unaffected by Wapal depletion (Additional file 13: Figure S11a), which was consistent with our microarray data (Additional file 14: Table S3). In addition, we verified that Wapal depletion resulted in decreased H3K27me3 at the *c*-*Fos* promoter (Additional file 13: Figure S11b). We also detected the presence of H3K27me3 at the Wapal-binding site, indicating that PRC2 activity is also present at this site distal to the *c*-*Fos* promoter. Surprisingly, Wapal depletion had minimal effect on H3K27me3 levels at this site, indicating that Wapal depletion did not cause global changes in H3K27me3 levels across the entire locus. Collectively, these data indicate that Wapal is required for Polycomb-mediated silencing of *c*-*Fos* expression.

**Fig. 7:**
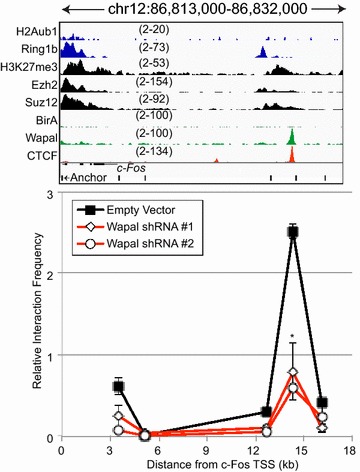
*Top panel* shows ChIP-seq tracks for PRC1 (H2Aub1, Ring1b), PRC2 (H3K27me3, Suz12, Ezh2), Wapal, CTCF, and BirA only as a negative control. Scale for each track is listed. Primers for 3C are shown below the gene, with the anchor primer labeled. *Bottom panel* normalized interaction frequency for different primers from cells infected with the empty vector or two different shRNAs to Wapal. *Asterisk* indicates a statistically significant (*p* value <0.05) reduction from empty vector

To address whether Wapal plays a role in regulating the interaction between this distal CRE and the *c*-*Fos* promoter, we utilized chromosomal conformational capture (3C) to directly measure the interaction frequency between the two. An area spanning 26 kb downstream of the *c*-*Fos* promoter was probed for interactions using an anchor primer near the TSS. An interaction was observed between the *c*-*Fos* promoter and the Wapal/CTCF distal CRE in cells infected with the empty vector, but no interactions were observed with primers flanking the Wapal-binding site (Fig. 7). Wapal depletion by two shRNAs results in decreased interaction between the 14.3 kb distal CRE and *c*-*Fos* promoter. Importantly, this result was observed 2 days following Wapal depletion, prior to cells displaying signs of differentiation. This indicates that Wapal is necessary for the distal CRE to interact with the *c*-*Fos* promoter. Collectively, these data imply that Wapal directly binds to a distal CRE within the *c*-*Fos* locus and assists with its recruitment to the TSS to mediate transcriptional repression through the PRC2 complex. Whether this is through participating in the chromatin looping directly or indirectly by influencing core cohesin subunits remains an open question.

## Discussion

Our work demonstrates that Wapal is critical to ESC pluripotency. This is consistent with a recent report that *Wapal*^−*/*−^ mice die early in embryogenesis [13]. Given our cell-cycle data (Fig. 1e), it appears that mitosis is unaffected 48 h after Wapal depletion and therefore likely not responsible for the loss of pluripotency we observe. The lack of cell-cycle changes in our experiments may result from redundancy by the separase complex, which can mediate sister chromatid separation independent of Wapal [4, 51]. A recent report using heterokaryons indicates that *Rad21* deletion may induce pluripotency factor expression and can improve reprogramming efficiency through increased expression of *c*-*Myc* [11]. Given the redundancy of the separase complex and the fact we use a depletion rather than genetic deletion strategy, our findings are most consistent with Wapal playing a critical role in pluripotency through modulating gene expression rather than altering the cell cycle. However, given these contradictory findings, further studies to understand Wapal and cohesin’s role in regulating pluripotency are required to separate effects on the cell cycle versus gene expression.

The biology of the cohesin complex has extensively been studied in mitosis, and the role of different subunits for sister chromatid cohesin has been used as a framework to understand their role during transcriptional regulation. Therefore, the canonical function of Wapal (off-loading cohesin) has been used as a model to understand Wapal’s role during interphase. Genetic deletion of *Wapal* [13] or a dominant-negative *Wapal* allele [12] increased bulk cohesin interactions with chromatin, as measured by fluorescence recovery after photobleaching (FRAP). The studies described herein used an alternative approach and identified >8000 genomic sites occupied by Wapal in ESCs by ChIP-seq. By overlaying these data with datasets for other core cohesin subunits, we demonstrate that Wapal co-occupies genomic sites with other cohesin proteins. In fact, genomic sites occupied by cohesin subunits with Wapal demonstrate slightly higher cohesin ChIP-tag density than sites occupied without Wapal, and there was minimal change in Rad21 chromatin occupancy after Wapal depletion (Fig. 3). This illustrates that Wapal does not appear to antagonize cohesin binding at these sites. Our data cannot exclude that Wapal affects the kinetics of cohesin occupancy at shared sites, given that we measure steady-state levels in a population of cells by ChIP-qPCR, rather than dynamic association rates which are measured by FRAP. However, we favor a model in which Wapal binds to specific genomic site in conjunction with other cohesin complex members. We favor this model because Wapal depletion phenocopies the biology of core cohesin subunits such as Rad21 or Smc3 in terms of (1) effects on ESC pluripotency after depletion; (2) chromatin occupancy; (3) transcriptome changes; (4) facilitating chromatin loops. At other sites within the genome not specifically occupied by Wapal, it likely off-loads cohesin to prevent chromatin condensation [13]. This highlights that Wapal’s role in regulating gene expression appears similar to core cohesin subunits in our studies.

Our data demonstrate that Wapal plays a critical role in regulating the epigenome, specifically PRC2-mediated H3K27me3 deposition. Whether this ultimately causes Wapal-depleted cells to lose pluripotency remains an unresolved issue. Deletion of either the catalytic subunit (Ezh2) or the core subunit (EED) required for PRC2 stability causes an early embryonic lethal phenotype in mice. However, both *Ezh2*^−*/*−^ and *EED*^−*/*−^ ESCs are pluripotent even though they lack PRC2 activity [52, 53]. In contrast, total H3K27me3 levels are unchanged after Wapal depletion by RNAi, implying that global PRC2 activity is preserved, a clear distinction with *EED*^−*/*−^ cells that lack H3K27me3. It is more likely that improper genomic localization of PRC2, rather than a complete loss of its activity, results in a loss of pluripotency. Polycomb-silenced genes are typically developmental regulators [54, 55], and therefore, derepression of even a handful of these loci would be sufficient to mediate a loss of pluripotency. For example, AP-1 family members such as c-Fos and c-Jun appear to be negative regulators of pluripotency, and depletion of either can enhance defined factor reprogramming [50].

**Fig. 8:**
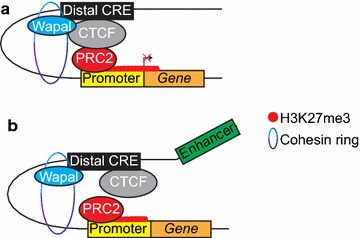
Two models to explain the role of Wapal and cohesin in regulating PRC2 targeting. **a** A CTCF-occupied distal CRE is responsible for recruiting PRC2 to mediate gene silencing. The recruitment is through a direct CTCF:Suz12 interaction [22]. In this model, the CTCF site would function as a mammalian PRE. **b** Polycomb complexes target all sites of transcription, and need to be “evicted” from the TSS by a transcriptional enhancer. CTCF prevents this by “blocking” enhancer access to gene. Cohesin facilitates the required DNA loop to stabilize the CTCF/gene chromatin interaction

Most intriguing is the role of Wapal in repression of *c*-*Fos* expression, with Wapal depletion associated with decreased H3K27me3 at the promoter and reduced interaction with a CTCF-occupied distal CRE. Given the high degree of overlap between Wapal and CTCF (97 %), we hypothesize two different models to explain how Wapal and CTCF may facilitate Polycomb targeting to effect gene silencing (Fig. 8). In the first (Fig. 8a), CTCF targets the PRC2 complex through a direct physical interaction with Suz12, similar to what occurs at *Igf2/H19* [22]. In this model, Wapal and cohesin work together to stabilize the resulting DNA loop, permitting gene silencing. In the second (Fig. 8b), CTCF blocks a distal transcriptional enhancer from accessing the gene promoter. In this model, Polycomb complexes bind to all sites of transcription and non-specifically silence them [21, 56]. Transcriptional enhancers are then required for Polycomb eviction, thereby facilitating gene expression [57]. The role of CTCF in enhancer blocking is well established and dependent on cohesin function [3]. While our data cannot distinguish between the two models, further studies utilizing genomic editing to delete different CREs coupled with genome-wide chromatin capture technologies (such as 4C, [58]) can be used to address this fundamental question.

Seminal studies in Drosophila regarding the role of cohesin in regulating gene expression and the epigenome demonstrate some similar but also disparate results from our own. In Drosophila, cohesin depletion decreased long-range interactions between a PRE and the *inv*-*en* gene complex [42], which is analogous to our results (Fig. 7). In contrast, work from Dr. Dale Dorsett’s laboratory [42, 59, 60] using the Drosophila wing imaginal disk as a model indicates that cohesin plays a fundamental role in sequestering the PRC1 complex at actively transcribed genes, thereby limiting its availability to bind to silenced genes. This sequestration is facilitated by a protein/protein interaction between cohesin and PRC1 in Drosophila [61]. While this exciting model may explain some of our observation, key distinctions between Drosophila and mammals likely prevent a direct comparison of cohesin’s function in regulating gene expression between these species. Specifically, cohesin genomic occupancy in Drosophila and mammals differs significantly [62]. In mammals, Wapal and cohesin predominantly bind chromatin in conjunction with CTCF [3, 63], mediated by a protein/protein interaction between CTCF and Stag2 [33]. In contrast, there are multiple insulator proteins in Drosophila beyond dCTCF (BEAF-32, Zw5, and Su(Hw)), which lack mammalian homologues and currently a paucity of evidence that these proteins interact with cohesin [64]. In fact, the Drosophila protein CP190 plays a critical role in mediating chromatin looping but lacks a mammalian homologue [44, 65]. Thus, it is likely that fundamental differences in the genomic localization of cohesin between Drosophila and mammals during interphase make direct comparisons between these species challenging.

## Conclusions

The core cohesin subunits play a critical role in transcriptional regulation by facilitating DNA loops that permit the interaction of distal *cis*-regulatory regions with gene promoters. However, the role of the cohesin off-loading protein Wapal in regulating gene expression has not been described. We show that Wapal depletion by RNAi induces the differentiation of ESCs, implying that it is required for pluripotency much like other core cohesin subunits. Wapal co-occupies genomic sites distal to genes in conjunction with other core cohesin subunits and CTCF, and at genomic sites it occupies Wapal does not appear to promote cohesin of off-loading. Wapal depletion causes a global derepression of Polycomb-silenced genes, although overall PcG activity remains unchanged. Lastly, Wapal facilitates the interaction between a distal CRE with the *c*-*Fos* promoter. Collectively, these data demonstrate that Wapal plays a critical role in PcG-mediated gene silencing.

## Methods

### ESC culture

Gelatin-adapted ESCs (129SVJ-dervied) were cultured as previously [25]. Bright-field images and alkaline phosphatase staining were performed as previously [24].

### Protein, RNA isolation, and quantitative PCR

Total RNA and total protein were collected as previously [25, 66]. If needed, RNA was further purified following manufacturer’s protocol (RNeasy Micro, Qiagen). cDNA conversions were performed using the iScript cDNA Synthesis kit (Biorad) according to manufacturer’s protocols. Quantitative real-time PCR (qPCR) was performed as previously [24, 25]. Primers used for reverse transcriptase-qPCR (RT-qPCR), ChIP-qPCR, and 3C are listed in Additional file 15: Table S4. The genomic regions of the primers (3C) or the amplified region (ChIP-qPCR) are indicated in Additional file 15: Table S4 (mm9).

### Western blots

Whole-cell extracts were prepared from cells by lysis in 50 mM Tris (pH 8.0), 10 % glycerol, 0.7 % NP-40 substitute (Sigma, 74385), 0.1 mM EDTA, 250 mM NaCl, 50 mM NaF, and 0.1 mM Na_3_VO_4_. Protease inhibitors were added to buffer as follows: 1:1000 DTT (Sigma, 646563), 5:1000 PMSF (Sigma, 93482-50ML-F), and 1:1000 Protease Inhibitor Cocktail (Sigma, P8340-5ML). Equal protein (10–50 μg) was run using standard SDS-PAGE separation techniques and blotted. Protein quantification was performed with Image J. All samples were normalized to a loading control (either GAPDH or total histone H3) and an empty vector or normal control.

### Antibodies

Smc3 (Abcam, Ab9263), Sall4 (Abcam, Ab29112), Wapal (Abcam, Ab70741), Scc1/Rad21 (Abcam, Ab992), Nanog (Millipore, AB5731), GAPDH-HRP (Santa Cruz, sc-25778), total H3 (Active Motif, 61278), H3K27me3 (Millipore, 07-449), H3K4me3 (Active Motif, 39916), anti-Brachyury (Santa Cruz, sc-17743), anti-v5 (Invitrogen, 46-0708), Streptavidin–HRP (Invitrogen, SA1007), anti-Suz12 (Santa Cruz, sc-46264), anti-Ezh2 (Millipore, 17-662), anti-Ring1b (Western Blot-Active Motif, 39664; ChIP-seq Abcam, Ab101273), anti-H2Aub1 (Cell Signaling, 8240).

### Lentiviral generation and RNAi experiments

RNAi/TRC consortium lentiviral constructs were obtained from either Open Biosystems or Sigma-Aldrich. The following constructs were utilized: pLKO.1 is the parental viral vector without shRNA. Sall4 (TRCN0000097821) was used as a positive control and can be rescued by the Sall4b cDNA [24], indicating that the loss of pluripotency is not secondary to off-target effects. Other lentiviruses used are against Wapal (TRCN0000178287, TRCN0000177268) or Smc3 (TRCN0000109007, TRCN0000160440). Lentiviruses were generated and ESCs infected as done previously [25]. Puromycin selection (4 μg/mL) was started after infection and continued daily for 2–6 days to select for cells with a high multiplicity of infection.

### Chromatin immunoprecipitation

Antibody-mediated ChIP and biotin-mediated ChIP (bioChIP) were performed similar to our previous work [25]. Primers used for ChIP-qPCR are indicated in Additional file 15: Table S4. Antibodies used are as above. A modified version of [67] was used for antibody-mediated ChIP on histones. Briefly, cells were cross-linked for 5 min at room temperature with 1 % fresh formaldehyde. Reaction was quenched with glycine (125 mM final concentration) for 5 min at room temperature. Cells were lysed in 1 % SDS lysis buffer (1 % SDS, 10 mM EDTA, 50 mM Tris–HCl pH 8.0, protease inhibitors). Chromatin was sonicated to a mean size of 300–500 bp. Lysed cells were cleared by centrifugation and supernatant diluted 1:20 with ChIP dilution buffer (0.01 % SDS, 1.1 % Triton X-100, 1.2 mM EDTA, 16.7 mM Tris–HCl, pH8.0, 167 mM NaCl, protease inhibitors). In total, 2–4 μg of antibodies were added and incubated overnight with constant rotation at 4 °C. Washed Dynal Protein A beads (Invitrogen) were added and incubated at 4 °C for an additional 90 min. Beads were isolated with a magnet and then washed with ChIP dilution buffer, low-salt buffer (0.1 % SDS, 1 % Triton X-100, 2 mM EDTA, 20 mM Tris–HCl pH 8.0, NaCl 150 mM), LiCl wash (250 mM LiCl, 0.5 % NP-40, 0.5 % deoxycholate, 1 mM EDTA, 10 mM Tris–HCl pH 8.0), and TE. DNA was eluted and de-cross-linked overnight at 65 °C in SDS elution buffer (1 % SDS, 10 mM EDTA, 50 mM Tris–HCl pH 8.0). The next day RNase A and proteinase K were added, and the DNA was precipitated in the presence of glycogen. DNA for downstream applications was quantitated by fluorometry (Qubit, Invitrogen), and equal DNA was used for each ChIP-qPCR and normalized to sheared, non-IP’d genomic DNA (input).

### Plasmid and cell line generation

The Wapal open reading frame was obtained from Open Biosystems and used as a template to perform site-directed mutagenesis to disrupt the third base pair of each codon within the shRNA complementary region, thus making it immune to the shRNA without altering the protein-coding sequence. A c-terminal v5 epitope tag was added to aid in detection. The cDNA was then cloned into the pPyCAG iH vector ([68], hygromycin resistance) for expression under a ubiquitous (CAG) promoter. CJ9 ESCs were electroporated with the linearized plasmid in the presence of hygromycin and individual clones isolated, as we have done previously [24]. All constructs were fully sequenced to ensure the coding sequence was correct. Expression of a tagged protein of the appropriate size was confirmed for each clone. The Wapal cDNA was cloned into the Flag/Bio expression vector and electroporated into BirA-expressing ESCs as we have done previously [24]. Individual colonies were expanded, and expression of a tagged, biotin-containing band of the correct size was verified by Western Blot with streptavidin and IP with streptavidin and blotting with anti-Wapal.

### Generation of Oct4:IRES:EGFP reporter ESC line

To generate the *Oct4:IRES:EGFP* reporter line used in Additional file 1: Figure S1A, a published guide RNA (gRNA) and plasmid were used to target CJ9 ESCs [27] and insert an *IRES:EGFP* cassette into the 3′ UTR of Oct4. Briefly, a guide RNA specific to the 3′ UTR of the *Oct4* locus (CTCAGTGATGCTGTTGATC) was cloned into the Cas9-expressing vector px459 [69]. A homology donor repair (HDR) vector containing an *IRES*:EGFP cassette flanked by homology regions from *Oct4* was obtained from Addgene (plasmid #48681) and co-transfected along with the guide RNA into 1–2 × 10^6^ wild-type ESCs. Transfected cells were selected with puromycin (2 μg/mL first 2 days only) and G418 (500 μg/mL daily) until single colonies appeared. Individual clones were isolated and cells that expressed GFP only in the pluripotent state were isolated, expanded, and used for subsequent experiments.

### ChIP-seq

Biotin-mediated Chromatin IP (bioChIP) was performed similar to previously [24, 25]. Briefly, 10–20 ng of ChIP DNA from two independently derived Flag/Bio Wapal clones, and control cells (expressing BirA) alone, along with an equal amount of sheared genomic DNA (input), were used for library generation (NEXTflex Chip-seq kit, Bioo Scientific) according to manufacturer’s protocol. Samples were sequenced on an Illumina HiSeq 2000 at Beijing Genomics Institute and obtained at least 50 million aligned reads per sample. For ChIP-seq on Wapal-depleted cells, a similar approach was used but cells were cross-linked and chromatin prepared 48 h post-infection. Library preparation was performed with NEB E7645 and sequenced on an Illumina NextSeq 500, 75 cycles, single end. The first 5 bp was trimmed from all reads due to lower Q30 scores, and then, the next 49 bp was used for alignment to recreate the analysis pipeline used for Wapal. After adaptor removal, reads were aligned to a reference genome (mm9) using Bowtie [70] with the following parameters (-e 70 -n 2 -k 1 -m 1), and then, peak calling was performed with MACS [71] using the default parameters (*p* value of 10^−5^). For each sample (input, BirA ChIP, Wapal ChIP #1 and #2), at least 50 million aligned reads were used for peak calling, and peak calling was performed by comparing experimental samples to input as a control (i.e., two-sided peak calling). Annotation of peaks was done using CisGenome [72]. Published datasets were obtained from the GEO Omnibus and are listed in Additional file 16: Table S2 for the following ChIP-seq datasets (Smc1a, Smc3, Rad21, CTCF, H3K27me3, Nanog, Stag1, Stag2, H2Aub1, Ring1b, Ezh2, and Suz12). All downloaded SRA-type files were analyzed using the same parameters as above. A file in.bed format with all Wapal peaks regions is included in Additional file 8: Table S1. A total of 266 genomic sites displayed significant biotinylation in the BirA-only-expressing cells, likely secondary to biotinylated histones, and are also included in Additional file 8: Table S1. We subtracted these regions from our subsequent analysis and identified a total of 8296 Wapal-binding sites between the two datasets. ChIP-seq tag pileup files were normalized using Haystack [73] when comparisons were made between different wiggle files.

### Transcriptome analysis

Total RNA was extracted using Trizol from ESCs infected with either empty vector (pLKO.1) or two, independent shRNAs to Wapal or Smc3. Two biological replicates for each shRNA or empty vector were used. Probes were generated and hybridized to Affymetrix Mouse Genome 430 2.0 arrays as previously [24]. CEL files were normalized using RMA [74] and subsequently processed using GenePattern [75] and GSEA [35] similar to our previous work using default parameters [24] unless otherwise indicated. For our initial GSEA, we used the C2:CGP group of datasets (v5) available through the Molecular Signature Database (MSigDB; http://www.broadinstitute.org/gsea/msigdb/collections.jsp).

### Cell-cycle analysis

ESCs were trypsinized, counted, and washed with PBS-/-. In 100 μL of PBS-/-, 0.5–1 × 10^6^ cells were resuspended, and 1.9 mL of cold 70 % ethanol was added in a dropwise fashion to fix cells. Cells were incubated at −20 °C for 30 min, spun down, and RNase digested at 37 °C for 30 min. Cells were stained with propidium iodide (PI, final concentration 10 μg/mL) for at least 5 min. Flow cytometry was performed and data was analyzed with FlowJo.

### Chromosome conformation capture (3C): modified from [76]

#### Library preparation

For each library generated, approximately 15 × 10^6^ mESC were fixed in a 1 % formaldehyde/10 % FBS solution for 10 min. Nuclei were isolated and digested with 1000U HaeIII (NEB R0108M) overnight at 37 °C while shaking. DNA was ligated with 7000 units of T4 DNA ligase (NEB M0202M) for 5 h at 16 °C. Samples were then de-cross-linked overnight at 65 °C. Following two phenol/chloroform extractions, the library was precipitated with a standard ethanol/sodium acetate protocol.

#### BAC preparation

Twenty micrograms of BAC RP23206I6 (CHORI) was digested with 1250U HaeIII overnight at 37 °C while shaking. Digested BAC DNA was phenol/chloroform extracted then ethanol precipitated. BAC was then ligated with 7000 units of T4 DNA ligase overnight in a 16 °C water bath. Ligated BAC template was phenol/chloroform extracted and ethanol precipitated. Template was resuspended in TE.

#### Determination of interaction frequency

Template and BAC loading were normalized by subtracting the CT of the internal primers from the CT of the test primers yielding ∆CT. The ∆CT from the BAC was then subtracted from ∆CT of the template to remove random interactions and PCR biases from the test primers. Interaction frequency is then determined by the value of 2^−(∆CT of template - ∆CT of BAC).

### Statistical analysis

Unless otherwise stated, *t* test comparisons were made and *p* values <0.05 were considered significantly different. All error bars are SEM derived from three experiments unless specifically stated otherwise.

### Genome-wide datasets

All microarray and ChIP-seq data are available in GEO Omnibus (GSE63325).

## Additional files

10.1186/s13072-016-0063-7 **A)** Cells expressing EGFP under the control of the *Oct4* locus were generated (see Materials and Methods section for details). GFP mean fluorescence intensity (MFI) was measured 2, 4, and 6 days post-infection. All data are normalized to the GFP MFI of cells infected with the empty vector on day 0, displayed on the y-axis. **B)** Wild-type (WT) or cells expressing a full-length Wapal cDNA immune to shRNA #2 were infected and bright field (10x, top panel) or alkaline phosphatase stained (4x, bottom panel) are shown 6 days post-infection. Domain structure of Wapal is shown to right of images. **C)** mRNA levels of different cohesin (Wapal and Smc3) and pluripotency (Nanog, Oct4) are shown. A linear scale of relative expression (compared to WT cells infected with the empty vector) is shown on the y-axis. * indicates a statistically significant reduction from the WT+empty vector control (p value<0.05). **D**) mRNA levels of differentiation markers. A Log_10_ scale (left) or linear (right) of relative expression (compared to WT cells infected with the empty vector) is shown on the y-axis. * indicates a statistically significant increase from the WT+empty vector control (p value<0.05). + indicates a statistically significant reduction in expression from WT+Wapal shRNA #2 (p-value<0.05). **E)** Western blots on cells six days after infection. Antibodies used are indicated on the right. E=empty vector, W= Wapal shRNA #2, WT= wild-type.
10.1186/s13072-016-0063-7 **A)**Western blot of either the parental (BirA) or cells expressing Flag/biotinylated (FB) version of Nanog or Wapal. Streptavidin HRP specifically recognizes the biotinylated versions of the proteins. * indicates nonspecific bands. **B)** Overlap between the ChIP-seq datasets derived from BirA cells and the two experimental cell lines. **C)** Five different Wapal sites identified by ChIP-seq were confirmed by ChIP-qPCR. All FB Wapal samples yielded statistically significant (*p* value <0.05) enrichment compared to input.
10.1186/s13072-016-0063-7 IGV screen captures of the *c-Myc* locus occupied by multiple members of the cohesin complex, including Wapal. Other loci tested in Figure S2c are shown in other figures (see below). For each graph, the genomic coordinates are shown below the x-axis. The y-axis represents the # of ChIP-seq tags recovered for a given genomic bin. The range for each track is displayed to the right of track name. A lower threshold of 2 was set for all tracks to minimize background. In all cases, the y-axis for BirA and Wapal is the same to demonstrate binding specificity.
10.1186/s13072-016-0063-7 The ChIP-seq tag densities of Smc3 or Smc1a were compared at genomic sites occupied with (Red) or without (Green) Wapal in a 2.5kb window around peak center. ChIP-seq tag densities for each Smc3 or Smc1a binding site within the genome are visualized as individual rows, with the color scale indicating a linear gradient from 0 to maximal tag values. Two different plots were generated based upon the binding with (left) or without (right) Wapal. Row order is from lowest binding to highest binding. # of sites used for each plot is indicated below.
10.1186/s13072-016-0063-7 **A)** Bright-field images of ESCs 48 hours post-infection with the empty vector or two shRNAs to Wapal. **B)** RT-qPCR to measure transcript levels 48hrs after Wapal depletion with two shRNAs. * indicates a statistically significant difference from empty vector (p value<0.05). **C)** Two gene sets recovered from gene set enrichment analysis that showed negative enrichment in the Wapal-depleted cells. NES=normalized enrichment scores, negative score indicates enrichment in the empty vector sample. **D)** Two independently derived lists of Polycomb target genes were used as gene sets and displayed significant enrichment with positive NES scores in Wapal-depleted samples, indicating that these genes are derepressed after Wapal depletion. **E**) Similar to (D), but using genes identified as PRC1 marked (H2Aub1) or PRC2 marked (H3K27me3) in their promoter.
10.1186/s13072-016-0063-7 All statistically significant altered gene sets from GSEA analysis after Wapal depletion.
10.1186/s13072-016-0063-7 **A)** The normalized ChIP-seq tag densities of Ring1b were compared at PcG-marked genes in cells infected with the empty vector (Black) or two separate shRNAs to Wapal (Red). X-axis is the distance in bp around TSS, and y-axis is the normalized tag #. Heat maps are similar to S4. A total of 1,455 PcG-marked genes were used for these analyses. **B)** Ring1b binding before (Black) or after Wapal depletion (Red) was measured at 1455 genes (same # as in A), which were either expressed at low (left) or high (right) levels. **C**) Similar to B, but genes where went down (left) or up (right) after depletion of Nanog or Oct4 in ESCs are shown.
10.1186/s13072-016-0063-7 Genomic sites occupied by Wapal.
10.1186/s13072-016-0063-7 Both (A) and (B) are similar to Figure 5, but only a subset of genes is used. **A)** 384 Polycomb-marked genes derepressed after Wapal depletion were used to measure the number of ChIP-seq tags recovered for either Wapal or the negative control BirA. **B)** For 384 genes, the relative binding at each gene is shown for Wapal and BirA alone samples. **C)** Smc3 binding at 1,455 PcG-marked genes. Smc3 control is an input sample. **D)** A total of 1,455 PcG-marked genes were used to measure number of ChIP-seq tags recovered for Smc3.
10.1186/s13072-016-0063-7 **A)** Log_2_ fold change in expression of six Polycomb-marked genes derepressed after Wapal depletion with two different shRNAs. Color scheme shown to right. **B)** IGV screen captures of two loci (Snai2 and Ppp1r3c) in which a combined Wapal-/CTCF-binding site is >2kb from the TSS, but also illustrates increased binding of Wapal around the TSS. Other aspects are similar to Figure S3.
10.1186/s13072-016-0063-7 Similar to (S8b), but for additional loci. A clear peak of Wapal binding is not present within 10kb of these four derepressed genes.
10.1186/s13072-016-0063-7 Protein levels after Wapal depletion of various histone marks and core components of the PRC1 and PRC2 complexes. Total H3 is used as a loading control. Quantification is described within Materials and Methods.
10.1186/s13072-016-0063-7 **A)** mRNA expression of Smc3, CTCF, and c-Fos 48 hours after Wapal depletion is shown. * indicates a statistically significant increased from empty vector (p value<0.05). **B)** ChIP-qPCR with an antibody to H3K27me3 after Wapal depletion at two genomic elements, the *c-Fos* promoter and a combined Wapal/CTCF site approximately 16kb downstream of the TSS. The genomic region is shown in Figure S3. * indicates statistically significant increase of empty vector over input (p value<0.05). + indicates statistically significant decrease of Wapal-depleted samples from empty vector (p value<0.05).
10.1186/s13072-016-0063-7 Normalized microarray expression data.
10.1186/s13072-016-0063-7 List of all primers used.
10.1186/s13072-016-0063-7 List of datasets, total # of reads, and # of aligned reads used.

## Abbreviations

ESC: embryonic stem cell
ChIP-seq: chromatin immunoprecipitation coupled with next-generation sequencing
PcG: Polycomb group
AML: acute myeloid leukemia
PRC1: Polycomb repressive complex 1
PRC2: Polycomb repressive complex 2
CRE: *cis*-regulatory elements
CdLS: Cornelia de Lange Syndrome
TF: transcription factor
RNAi: RNA interference
PRE: Polycomb response element
shRNAs: short hairpin RNAs
IRES: internal ribosomal entry site
EGFP: enhanced green fluorescent protein
MFI: mean fluorescent intensity
PI: propidium iodide
FL: full-length
RT: reverse transcription
qPCR: quantitative PCR
GSEA: gene set enrichment analysis
H2AUb1: ubiquitination of histone 2A
H3K27me3: trimethylation of histone 3 on lysine 27
TSS: transcriptional start site
3C: chromosomal conformational capture
AP: alkaline phosphatase
MOI: multiplicity of infection
NES: normalized enrichment scores
FDR: false discovery rate
WT: wild-type
