# Supplementary figures and images for: The cohesin-associated protein Wapal is required for proper Polycomb-mediated gene silencing
Source: Epigenetics Chromatin. 2016 Apr 15;9:14. doi: 10.1186/s13072-016-0063-7 (PMC4832553; doi:10.1186/s13072-016-0063-7)

Figure S1

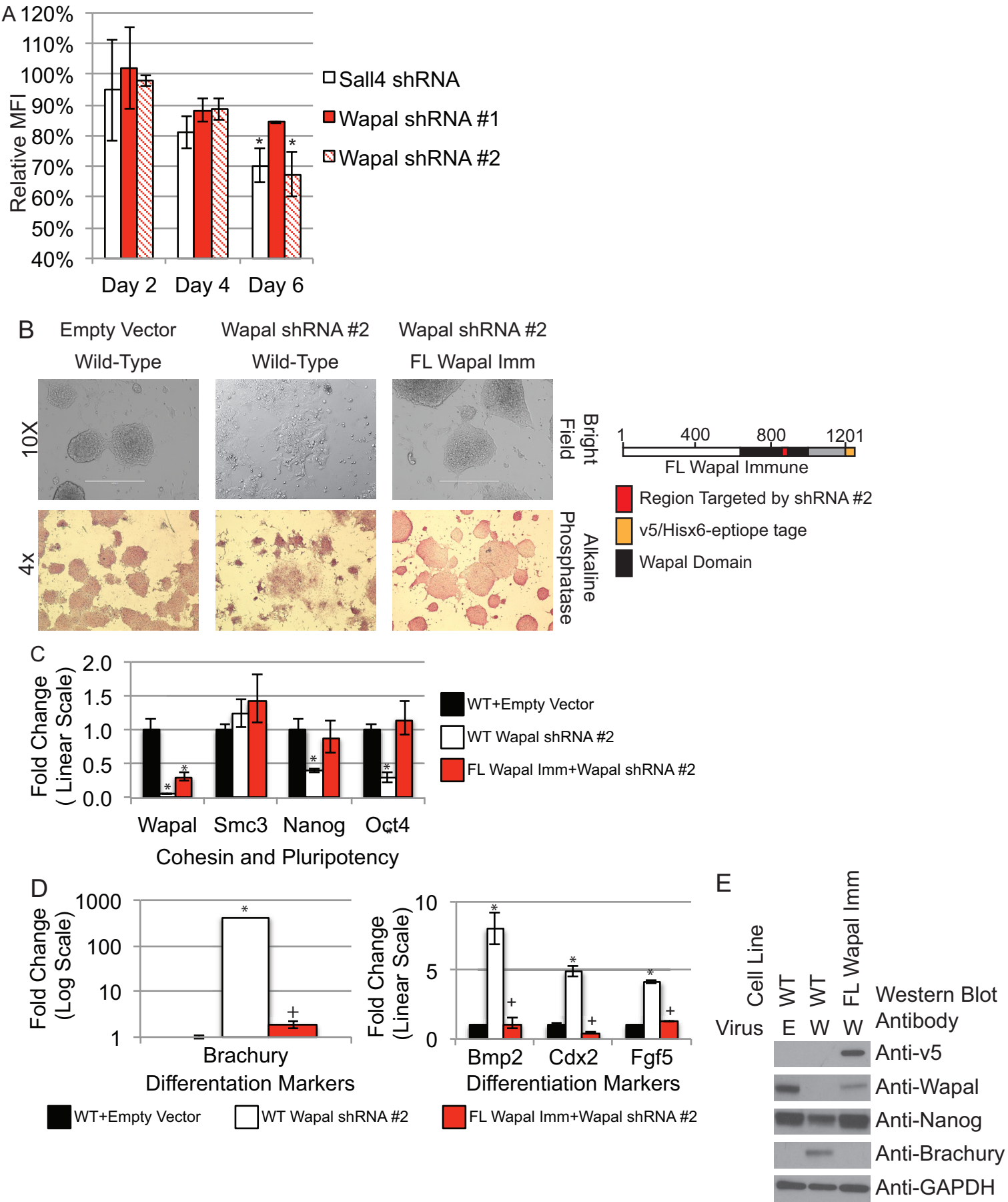

Supplement: Supplementary file 1 — 10.1186/s13072-016-0063-7 A) Cells expressing EGFP under the control of the Oct4 locus were generated (see Materials and Methods section for details). GFP mean fluorescence intensity (MFI) was measured 2, 4, and 6 days post-infection. All data are normalized to the GFP MFI of cells infected with the empty vector on day 0, displayed on the y-axis. B) Wild-type (WT) or cells expressing a full-length Wapal cDNA immune to shRNA #2 were infected and bright field (10x, top panel) or alkaline phosphatase stained (4x, bottom panel) are shown 6 days post-infection. Domain structure of Wapal is shown to right of images. C) mRNA levels of different cohesin (Wapal and Smc3) and pluripotency (Nanog, Oct4) are shown. A linear scale of relative expression (compared to WT cells infected with the empty vector) is shown on the y-axis. * indicates a statistically significant reduction from the WT+empty vector control (p value<0.05). D) mRNA levels of differentiation markers. A Log10 scale (left) or linear (right) of relative expression (compared to WT cells infected with the empty vector) is shown on the y-axis. * indicates a statistically significant increase from the WT+empty vector control (p value<0.05). + indicates a statistically significant reduction in expression from WT+Wapal shRNA #2 (p-value<0.05). E) Western blots on cells six days after infection. Antibodies used are indicated on the right. E=empty vector, W= Wapal shRNA #2, WT= wild-type. [file 13072_2016_63_MOESM1_ESM.pdf]

Figure S2

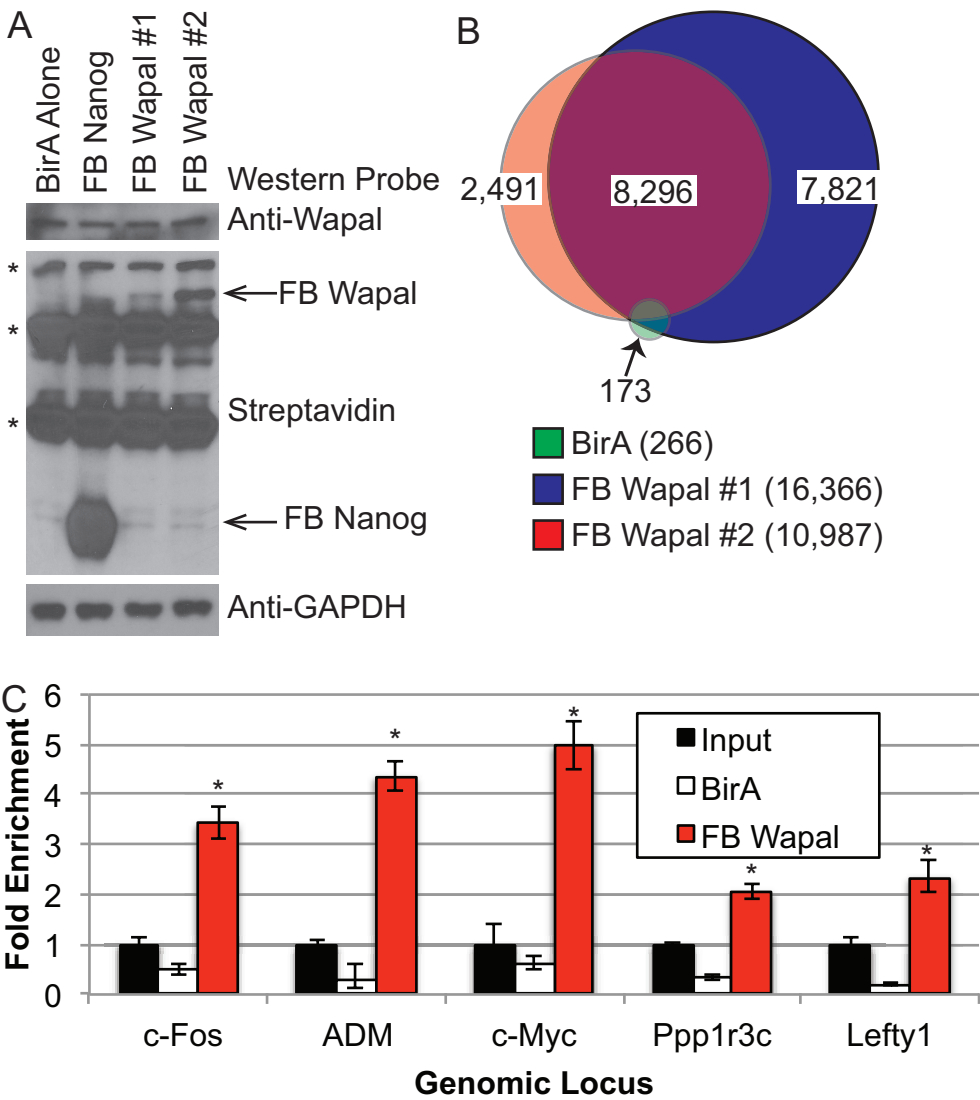

Supplement: Supplementary file 2 — 10.1186/s13072-016-0063-7 A)Western blot of either the parental (BirA) or cells expressing Flag/biotinylated (FB) version of Nanog or Wapal. Streptavidin HRP specifically recognizes the biotinylated versions of the proteins. * indicates nonspecific bands. B) Overlap between the ChIP-seq datasets derived from BirA cells and the two experimental cell lines. C) Five different Wapal sites identified by ChIP-seq were confirmed by ChIP-qPCR. All FB Wapal samples yielded statistically significant (p value <0.05) enrichment compared to input. [file 13072_2016_63_MOESM2_ESM.pdf]

Figure S3

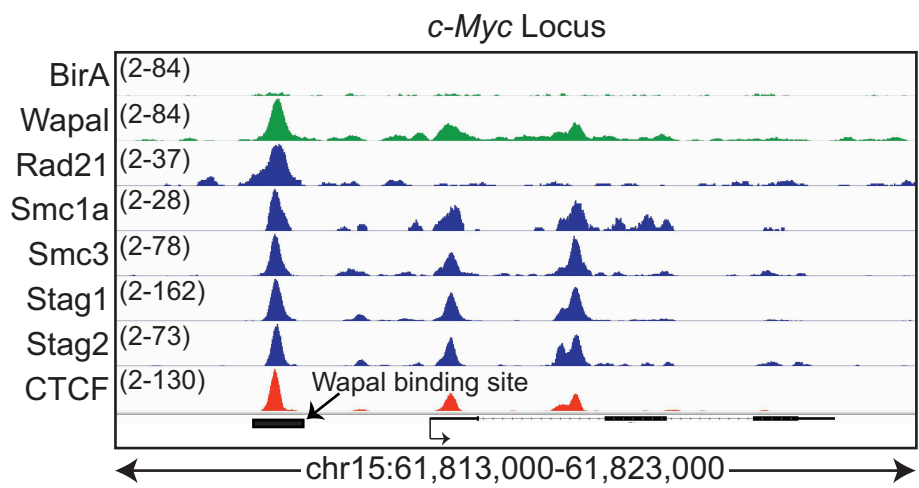

Supplement: Supplementary file 3 — 10.1186/s13072-016-0063-7 IGV screen captures of the c-Myc locus occupied by multiple members of the cohesin complex, including Wapal. Other loci tested in Figure S2c are shown in other figures (see below). For each graph, the genomic coordinates are shown below the x-axis. The y-axis represents the # of ChIP-seq tags recovered for a given genomic bin. The range for each track is displayed to the right of track name. A lower threshold of 2 was set for all tracks to minimize background. In all cases, the y-axis for BirA and Wapal is the same to demonstrate binding specificity. [file 13072_2016_63_MOESM3_ESM.pdf]

**Figure S4**

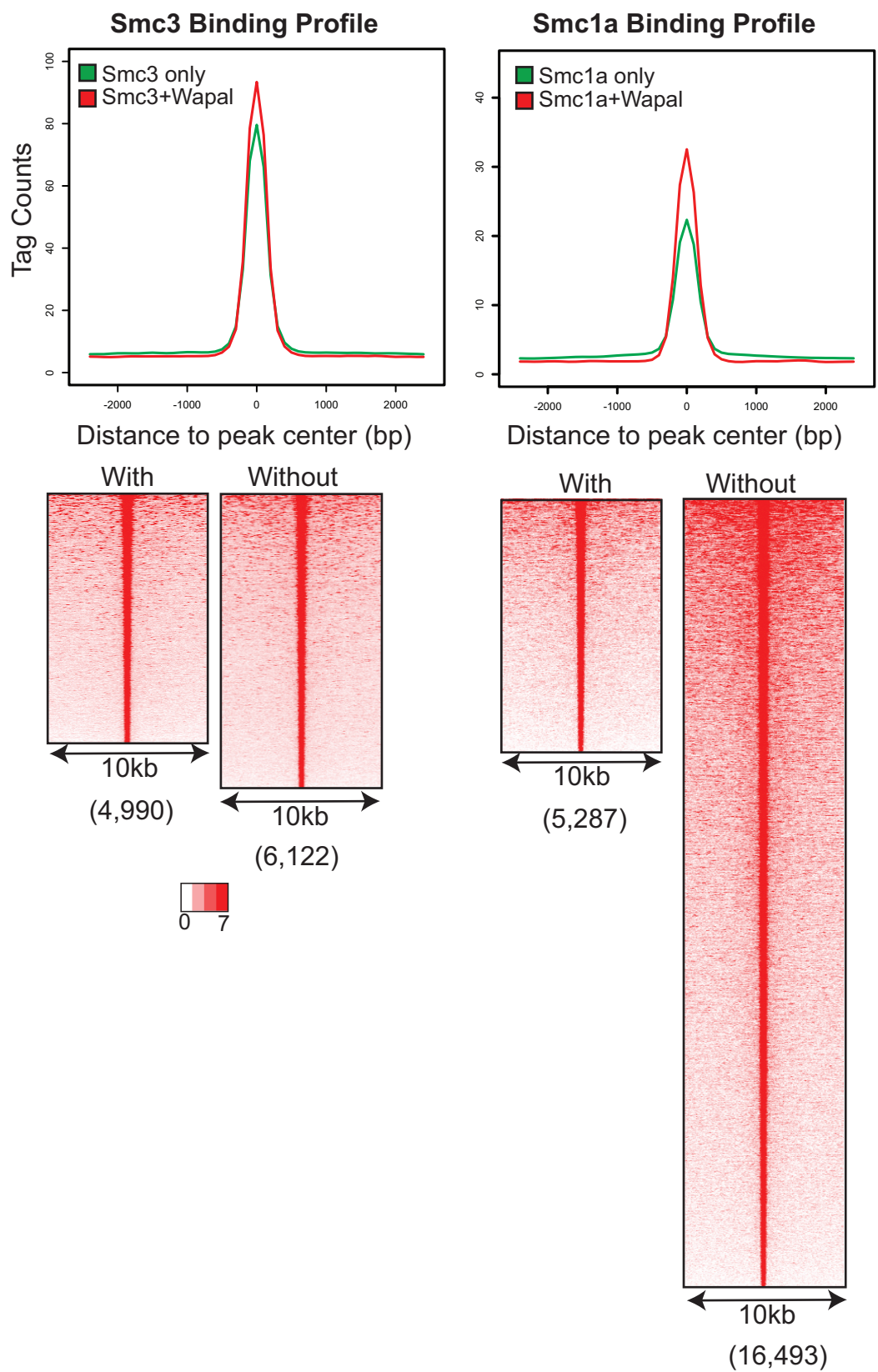

Supplement: Supplementary file 4 — 10.1186/s13072-016-0063-7 The ChIP-seq tag densities of Smc3 or Smc1a were compared at genomic sites occupied with (Red) or without (Green) Wapal in a 2.5kb window around peak center. ChIP-seq tag densities for each Smc3 or Smc1a binding site within the genome are visualized as individual rows, with the color scale indicating a linear gradient from 0 to maximal tag values. Two different plots were generated based upon the binding with (left) or without (right) Wapal. Row order is from lowest binding to highest binding. # of sites used for each plot is indicated below. [file 13072_2016_63_MOESM4_ESM.pdf]

Figure S5

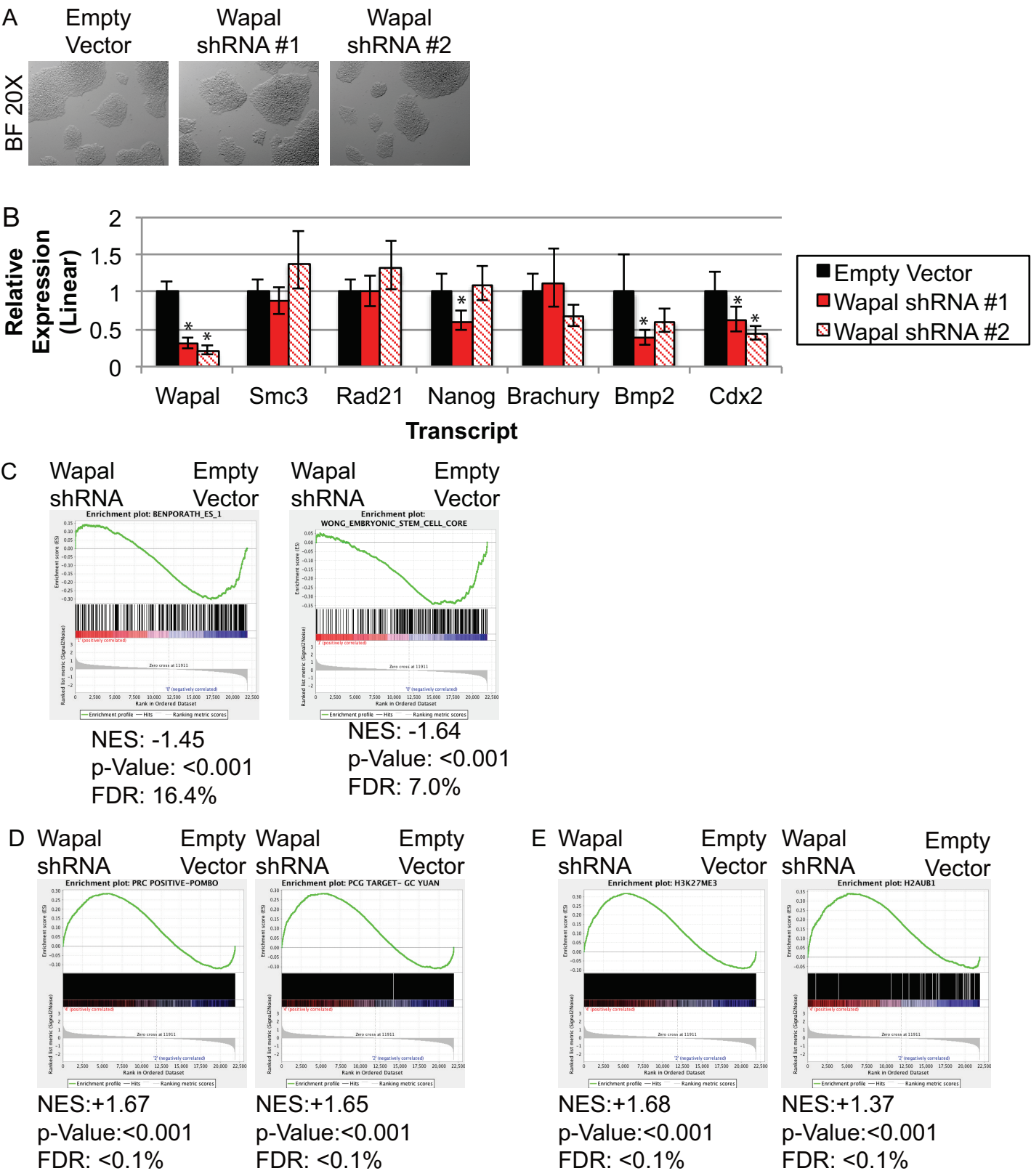

Supplement: Supplementary file 5 — 10.1186/s13072-016-0063-7 A) Bright-field images of ESCs 48 hours post-infection with the empty vector or two shRNAs to Wapal. B) RT-qPCR to measure transcript levels 48hrs after Wapal depletion with two shRNAs. * indicates a statistically significant difference from empty vector (p value<0.05). C) Two gene sets recovered from gene set enrichment analysis that showed negative enrichment in the Wapal-depleted cells. NES=normalized enrichment scores, negative score indicates enrichment in the empty vector sample. D) Two independently derived lists of Polycomb target genes were used as gene sets and displayed significant enrichment with positive NES scores in Wapal-depleted samples, indicating that these genes are derepressed after Wapal depletion. E) Similar to (D), but using genes identified as PRC1 marked (H2Aub1) or PRC2 marked (H3K27me3) in their promoter. [file 13072_2016_63_MOESM5_ESM.pdf]

**Figure S6**

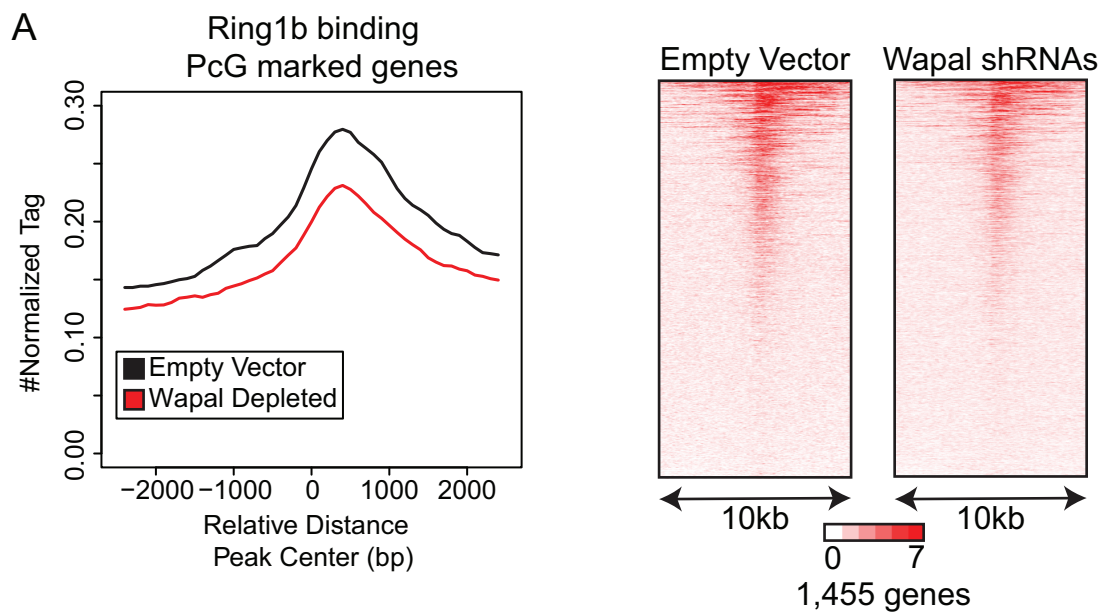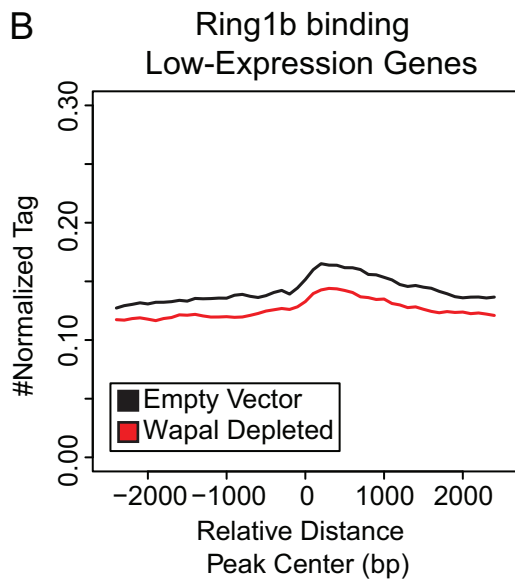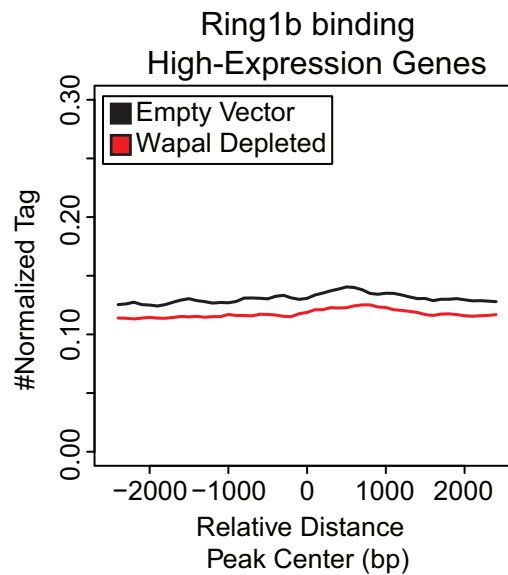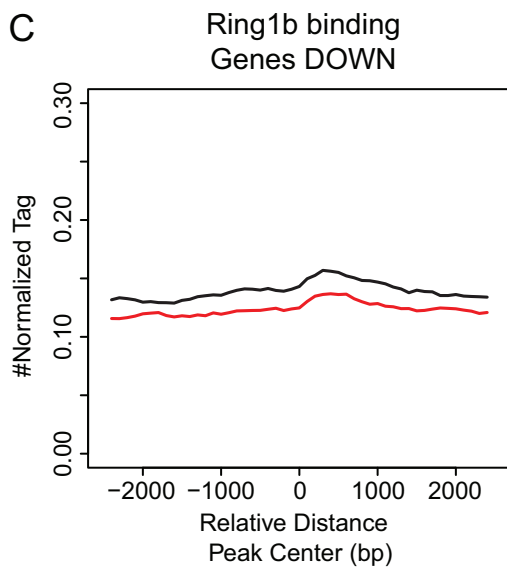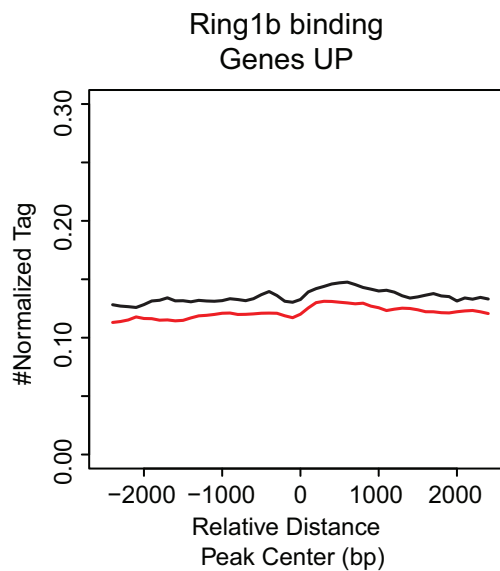

Supplement: Supplementary file 7 — 10.1186/s13072-016-0063-7 A) The normalized ChIP-seq tag densities of Ring1b were compared at PcG-marked genes in cells infected with the empty vector (Black) or two separate shRNAs to Wapal (Red). X-axis is the distance in bp around TSS, and y-axis is the normalized tag #. Heat maps are similar to S4. A total of 1,455 PcG-marked genes were used for these analyses. B) Ring1b binding before (Black) or after Wapal depletion (Red) was measured at 1455 genes (same # as in A), which were either expressed at low (left) or high (right) levels. C) Similar to B, but genes where went down (left) or up (right) after depletion of Nanog or Oct4 in ESCs are shown. [file 13072_2016_63_MOESM7_ESM.pdf]

**Figure S7**

**A** Wapal Binding at derepressed PcG marked genes

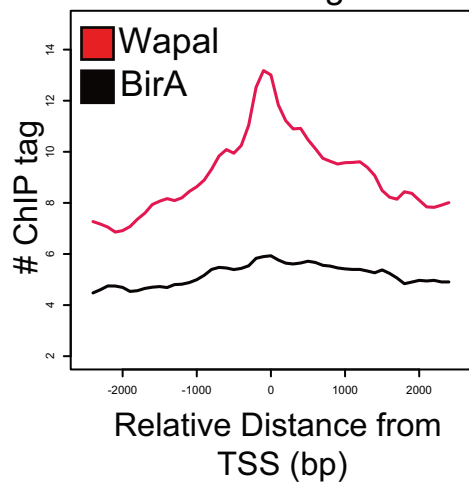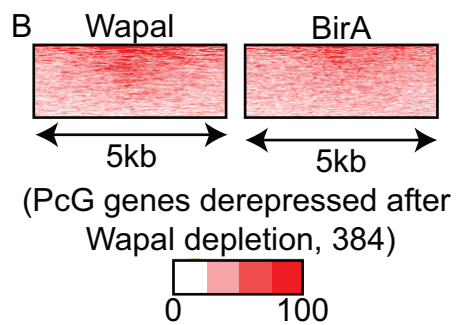

**C** Smc3 binding at PcG marked genes

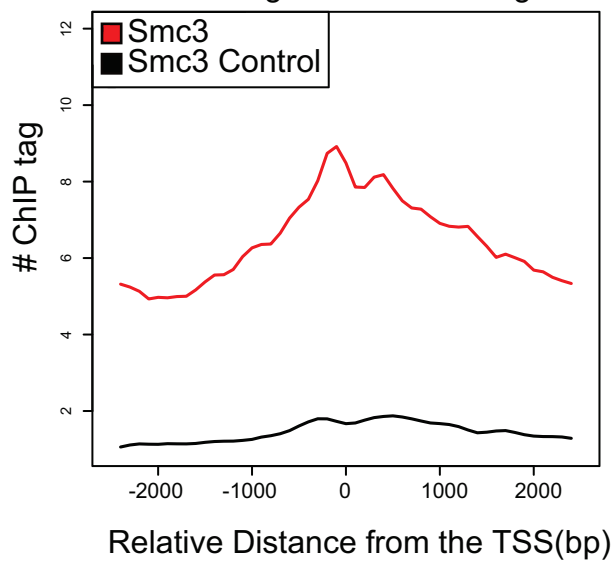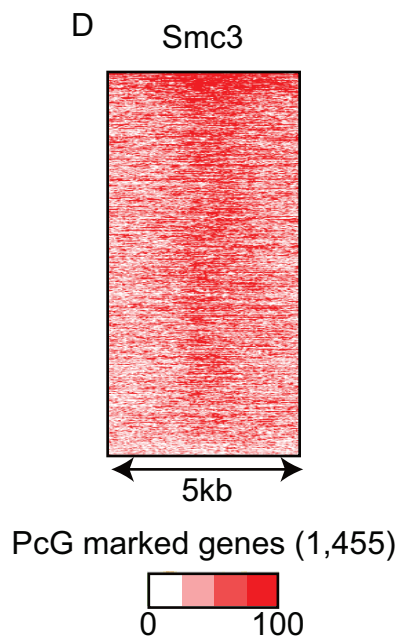

Supplement: Supplementary file 9 — 10.1186/s13072-016-0063-7 Both (A) and (B) are similar to Figure 5, but only a subset of genes is used. A) 384 Polycomb-marked genes derepressed after Wapal depletion were used to measure the number of ChIP-seq tags recovered for either Wapal or the negative control BirA. B) For 384 genes, the relative binding at each gene is shown for Wapal and BirA alone samples. C) Smc3 binding at 1,455 PcG-marked genes. Smc3 control is an input sample. D) A total of 1,455 PcG-marked genes were used to measure number of ChIP-seq tags recovered for Smc3. [file 13072_2016_63_MOESM9_ESM.pdf]

Figure S8

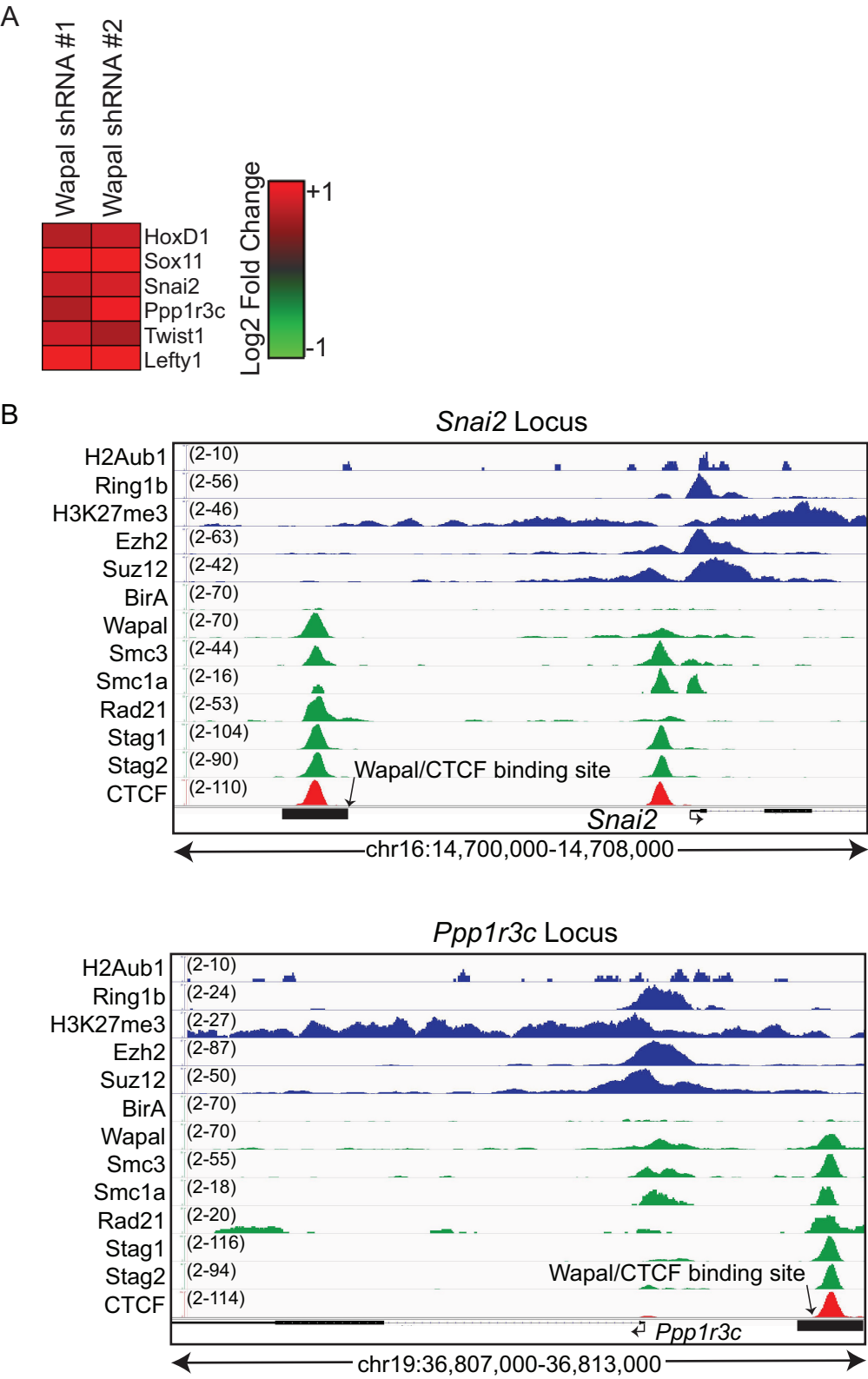

Supplement: Supplementary file 10 — 10.1186/s13072-016-0063-7 A) Log2 fold change in expression of six Polycomb-marked genes derepressed after Wapal depletion with two different shRNAs. Color scheme shown to right. B) IGV screen captures of two loci (Snai2 and Ppp1r3c) in which a combined Wapal-/CTCF-binding site is >2kb from the TSS, but also illustrates increased binding of Wapal around the TSS. Other aspects are similar to Figure S3. [file 13072_2016_63_MOESM10_ESM.pdf]

Figure S9

*HoxD1* Locus

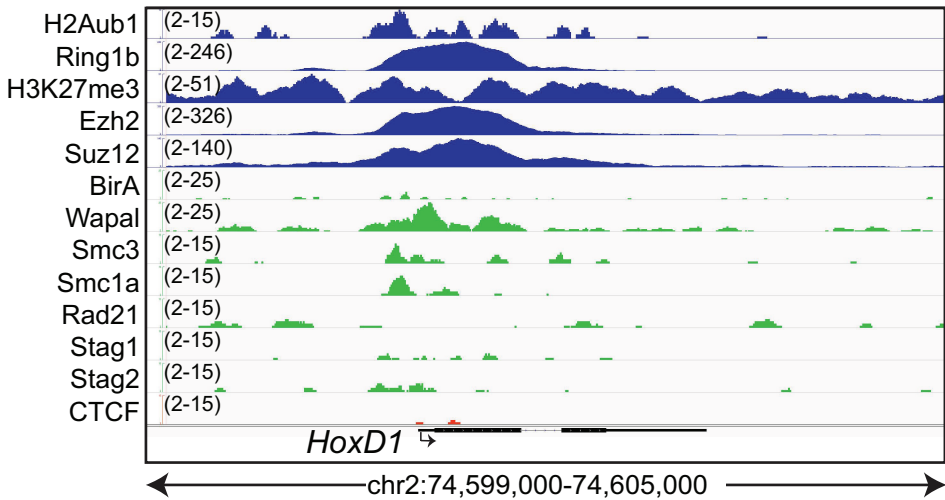

*Sox11* Locus

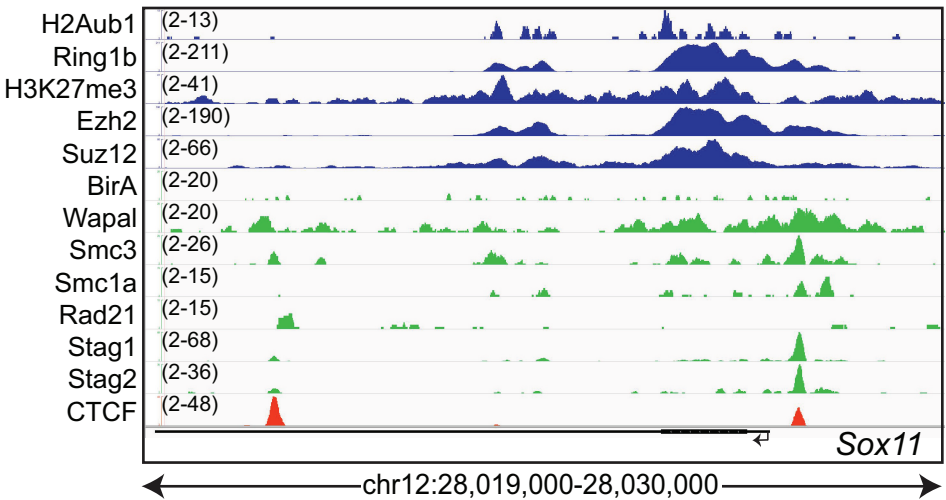

*Twist1* Locus

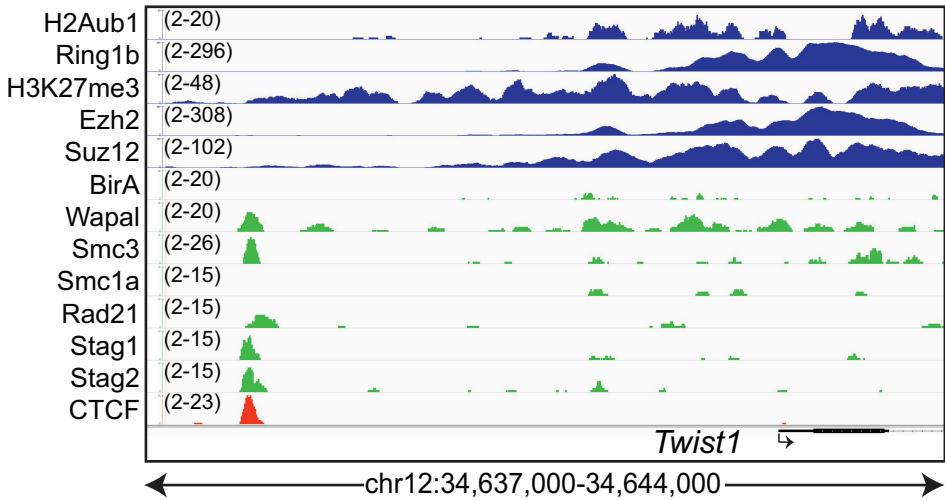

*Lefty1* Locus

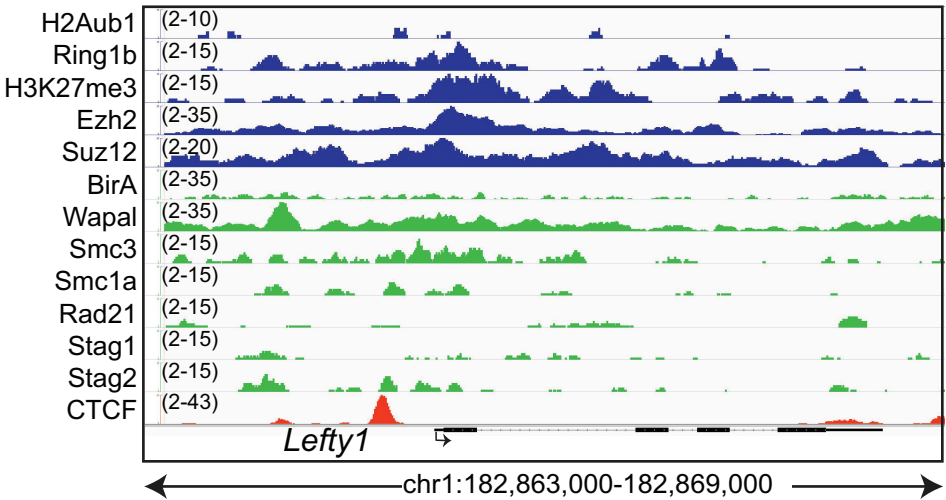

Supplement: Supplementary file 11 — 10.1186/s13072-016-0063-7 Similar to (S8b), but for additional loci. A clear peak of Wapal binding is not present within 10kb of these four derepressed genes. [file 13072_2016_63_MOESM11_ESM.pdf]

Figure S10

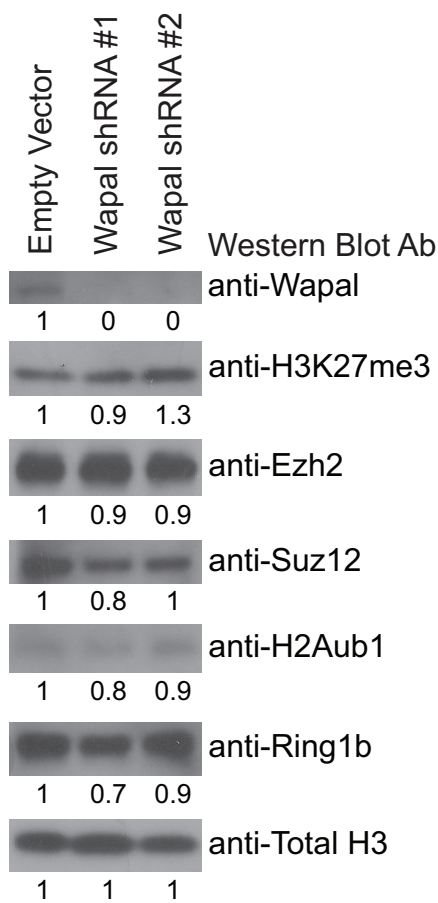

Supplement: Supplementary file 12 — 10.1186/s13072-016-0063-7 Protein levels after Wapal depletion of various histone marks and core components of the PRC1 and PRC2 complexes. Total H3 is used as a loading control. Quantification is described within Materials and Methods. [file 13072_2016_63_MOESM12_ESM.pdf]

**Figure S11**

**A**

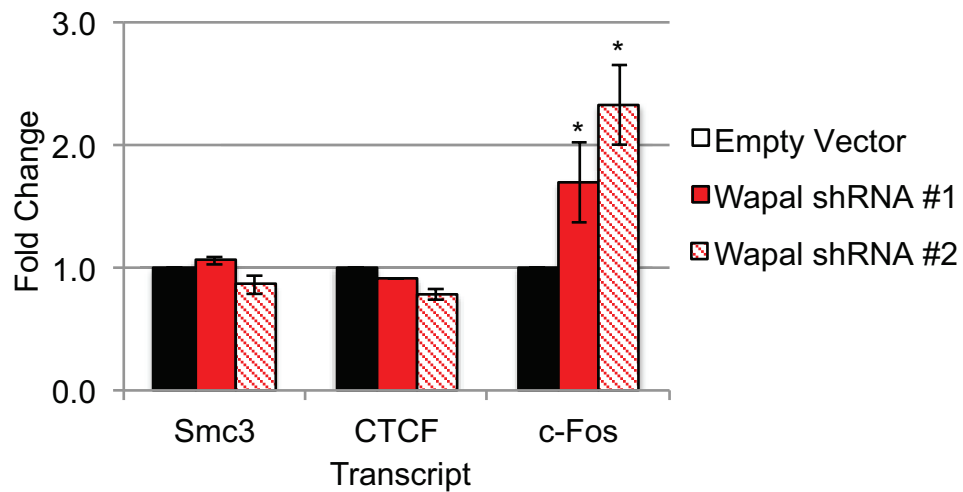

**B**

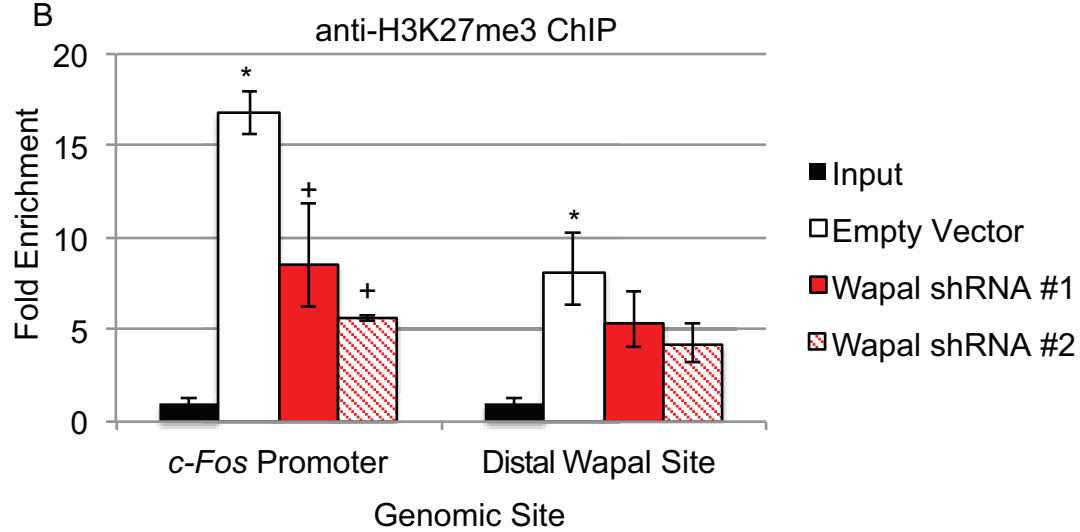

Supplement: Supplementary file 13 — 10.1186/s13072-016-0063-7 A) mRNA expression of Smc3, CTCF, and c-Fos 48 hours after Wapal depletion is shown. * indicates a statistically significant increased from empty vector (p value<0.05). B) ChIP-qPCR with an antibody to H3K27me3 after Wapal depletion at two genomic elements, the c-Fos promoter and a combined Wapal/CTCF site approximately 16kb downstream of the TSS. The genomic region is shown in Figure S3. * indicates statistically significant increase of empty vector over input (p value<0.05). + indicates statistically significant decrease of Wapal-depleted samples from empty vector (p value<0.05). [file 13072_2016_63_MOESM13_ESM.pdf]
